# Transcriptomic analysis of glucosidase II beta subunit (GluIIß) knockout A549 cells reveals its roles in regulation of cell adhesion molecules (CAMs) and anti-tumor immunity

**DOI:** 10.1186/s12864-023-09888-z

**Published:** 2024-01-20

**Authors:** Worapong Khaodee, Guo Xiyuan, Moe Thi Thi Han, Chatchai Tayapiwatana, Sawitree Chiampanichayakul, Songyot Anuchapreeda, Ratchada Cressey

**Affiliations:** 1 Department of Medical Technology, Faculty of Associated Medical Sciences, Chiang Mai University, Chiang Mai, Thailand; 2 Public Experimental Technology Center School of Basic Medical Sciences, Southwest Medical University, Luzhou, 646000 China; 3 Cancer Research Unit, Department of Medical Technology, Faculty of Associated Medical Sciences, Chiang Mai University, Chiang Mai, Thailand

**Keywords:** Glucosidase II beta subunit, PRKCSH, Transcriptomic analysis, Non-small cell lung cancers (NSCLCs), Cell adhesion molecules (CAMs), T cell

## Abstract

Glucosidase II beta subunit (GluIIß), encoded from *PRKCSH*, is a subunit of the glucosidase II enzyme responsible for quality control of N-linked glycoprotein folding and suppression of GluIIß led to inhibitory effect of the receptor tyrosine kinase (RTKs) activities known to be critical for survival and development of cancer. In this study, we investigated the effect of GluIIß knockout on the global gene expression of cancer cells and its impact on functions of immune cells. GluIIß knockout lung adenocarcinoma A549 cell line was generated using CRISPR/Cas9-based genome editing system and subjected to transcriptomic analysis. Among 23,502 expressed transcripts, 1068 genes were significantly up-regulated and 807 genes greatly down-regulated. The KEGG enrichment analysis showed significant down-regulation of genes related extracellular matrix (ECM), ECM-receptor interaction, cytokine-cytokine receptor interaction and cell adhesion molecules (CAMs) in GluIIß knockout cells. Of 9 CAMs encoded DEG identified by KEGG enrichment analysis, real time RT-PCR confirmed 8 genes to be significantly down-regulated in all 3 different GluIIß knockout clones, which includes cadherin 4 (CDH4), cadherin 2 (CDH2), versican (VCAN), integrin subunit alpha 4 (ITGA4), endothelial cell-selective adhesion molecule (ESAM), CD274 (program death ligand-1 (PD-L1)), Cell Adhesion Molecule 1 (CADM1), and Nectin Cell Adhesion Molecule 3 (NECTIN3). Whereas PTPRF (Protein Tyrosine Phosphatase Receptor Type F) was significantly decreased only in 1 out of 3 knockout clones. Microscopic analysis revealed distinctively different cell morphology of GluIIβ knockout cells with lesser cytoplasmic and cell surface area compared to parental A549 cells and non-targeted transfected cells.

Further investigations revealed that Jurkat E6.1 T cells or human peripheral blood mononuclear cells (PBMCs) co-cultured with GluIIß knockout A549 exhibited significantly increased viability and tumor cell killing activity compared to those co-cultured with non-target transfected cells. Analysis of cytokine released from Jurkat E6.1 T cells co-cultured with GluIIß knockout A549 cells showed significant increased level of angiogenin and significant decreased level of ENA-78. In conclusion, knockout of GluIIß from cancer cells induced altered gene expression profile that improved anti-tumor activities of co-cultured T lymphocytes and PBMCs thus suppression of GluIIß may represent a novel approach of boosting anti-tumor immunity.

**Supplementary Information:**

The online version contains supplementary material available at 10.1186/s12864-023-09888-z.

## Introduction

The immune system plays a dual role in cancer development. On one hand, the immune system targets and destroys malignant cells. On the other hand, immune system-mediated inflammation regulates numerous cell functions, which in turn inhibits the antitumor response, and influences subsequent treatment [1]. These immune responses are influenced by cell surface receptor and adhesion molecules, which are a class of glycoproteins on the cell membrane that are involved in the binding of cell to cell, to the extracellular matrix or to chemokines, and directly linked to tumorigenesis, and metastasis of tumor cells [2]. Glycoproteins consist of two main types, N-glycosylated and O-glycosylated, which differed in the types of their sugar–protein linkage. N-glycoproteins account for most receptors and adhesion molecules on the cell surface [3, 4]. Evidence suggests that alterations of N-glycosylation of these cell surface and cell adhesion proteins can change their biological activities including their cellular localization and signaling activities, affecting not only the growth and survival of cancer cells [5], but also the behaviors of immune checkpoint inhibitors [6]. It has been established that programmed cell death ligand 1 (PD-L1) requires N-linked glycosylation to maintain its stability, surface localization and interaction with its cognate receptor, programmed cell death protein 1 (PD-1), and this in turn promotes evasion of T-cell function [7, 8].

Glucosidase II beta subunit (GluIIß) is an ER-resident protein encoded from the *PRKCSH* gene and functions as a beta subunit of glucosidase II enzyme involved in the regulating of post-translation modification of N-linked glycoproteins [9]. This group of proteins play important roles in various biological processes, including cell-to-cell interaction, growth, differentiation, and programmed cell death [10]. Glucosidase II is responsible for the sequential removal of two glucose molecules during the folding of N-linked glycoproteins. The first glucose removal initiates the interaction of newly synthesized proteins to ER chaperone that help with protein folding and the later glucose removal permits their release from the ER. Only correctly folded proteins are allowed to leave the ER and transported to their designated cellular compartment to function while misfolded proteins are retained and subsequently degraded. GluIIß expression was reported to be increased in human tumor tissues [11, 12] and inhibition of its activity or expression led to the induction of autophagy and/or apoptosis [13, 14]. Tumor cells are constantly facing ER stress that occurred through the accumulation of misfolded proteins and/or the disruption of glycoprotein quality control. Therefore, increase expression of GluIIß in tumor cells is believed to help keeping up with an increase demand of N-linked glycoproteins within the cells while still maintaining ER homeostasis, which the overall purpose is to promote tumor survival and expansion.

In this study, RNA-Seq was used to evaluate the expression profile of GluIIß knockout A549 cells compared to non-target transfected cells. The analysis of enrichment pathways using GO and KEGG identified that GluIIß knockout greatly suppressed expression of extracellular matrices (ECM) and cell adhesion molecules (CAMs) that play an important role in modulating anti-tumor immunity. Further characterization revealed that this altered gene expression profile in GluIIß knockout cells improved viability and tumor lysing activity of Jurkat T lymphocytes and peripheral blood mononuclear cells (PBMCs). These data suggests that the increased level of GluIIß in tumor cells may serve as a mean for these cells to escape from immune surveillance thus suppression of GluIIß may represent as a novel approach of revitalizing anti-tumor immunity.

## Material and methods

### Cell line

A549 (adenocarcinomic human alveolar basal epithelial cells) obtained from American Tissue Culture Collection (ATCC) and Cas-9 stable transfected A549 was purchased from Ubigene Biosciences. A549 is an established cell line frequently used as a model for non-small cell lung cancers (NSCLCs), which is a common type of lung cancer make up about 85% of all the cases [15].

These cells were maintained in DMEM supplemented with 10% fetal bovine serum (FBS) (v/v), 100 units/ml penicillin and 100 µg/ml streptomycin (Gibco-Thermo Fisher Scientific, (Massachusetts, USA). Jurkat E6.1 cell line (human T cell lymphoblast-like cell line) kindly provided by Prof. Kasinrerk [16] were maintained in RPMI 1640, with the same supplementations as DMEM.

### Knockout of GluIIß using CRISPR/Cas9-mediated genome editing

A GluIIß knockout A549 cells was established by CRISPR/ Cas9-mediated genome editing from Ubigene (Ubigene Biosciences Co., Ltd., Guangdong, China). Caspase 9 stably expressed A549 cells (A549-cas9) was transfected with plasmid harboring guide RNA specific to human *PRKCSH* gene (YKO-RP003-*hPRKCSH* gRNA1). A549-cas9 cells were also transfected with plasmids carrying scramble gRNA (YKO-RP003-Scramble gRNA) as non-target transfected control cells.

Briefly, about 5 x 10^5^ cells/well of A549-cas9 stable cell line were seeded and cultured in a six well tissue culture plate overnight. 12.5 µl of (2 μg) of *hPRKCSH* gRNA plasmids or scramble gRNA plasmids diluted in 120 µl Opti-MEM media (Gibco, Life Technologies, Ltd., Paisley, UK) was mixed with 6 µl of HilyMax transfection reagent (H357, Dojindo Molecular Technologies Inc, Maryland, USA) and incubated for 15 minutes at room temperature. Afterward, the plasmid gRNA/ HilyMax transfection reagent complex was added dropwise with gentle swirling into cultured cells and incubated for 4 hours before replacing the cultured media with fresh media. Forty-eight hours later, cells were cultured in 1.2 µg/ml puromycin containing media for 3 weeks. Colonies of surviving cells were individually picked and expanded into larger vessels before subjecting to further tests. The knockout of GluIIß gene expression was confirmed by subjecting the surviving clones to the verification by Western blot analysis.

### Transcriptomic analysis of GluIIß knockout cells by RNA sequencing and data analysis

After verification of GluIIß knockout, transfected cells were harvested and subjected to total RNA extraction using NucleoSpin RNA Plus (Machery-Nagel), a kit for RNA purification with DNA removal column. The purity of extracted RNA from of GluIIß knockout cells and non-targeted transfected cells were verified to ensure that the OD260/280 and OD260/230 ratios were ≥ 1.8 before submitting to RNA sequencing via the next-generation sequencing technique (NGS) (Illumina NovaSeq 6000 Sequencing System) with NovogeneAIT (Genomics Singapore Pte Ltd, Singapore), in which the quality of RNA was then further assessed by agarose gel electrophoresis and the 2100 Bioanalyzer Instument (Agilent Biotechnology). The single-stranded messenger RNAs (mRNAs) were selectively captured and converted to complementary DNA (cDNA) for library preparation. The Illumina platform was then used for a paired ended 150 base-pair sequencing strategy (short-read) to sequence the cDNA libraries, which offer the whole transcriptomes analysis with the data output of ≥ 50 million read pairs per sample.

The transcripts with log2 fold change ≥ 2 and *p*-value ≤ 0.05 were considered as significantly upregulated, transcripts with log2 fold change ≤ −2 and *p*-value ≤0.05 were considered as significantly down regulated. The RNA sequencing data were also subjected to functional analysis. The cluster profiler [17] software for enrichment analysis, including GO (Gene Ontology) enrichment, KEGG (Kyoto Encyclopedia of Genes and Genomes) enrichment, Reactome enrichment and DisGeNET database enrichment, was used to identify the biological functions or pathways significantly associated with differential expressed genes.

### Isolation of human peripheral blood mononuclear cells (PBMCs)

PBMCs were isolated from healthy volunteers using HiSepTM LSM (HiMedia Laboratories). 2.5 ml of HiSep™ LSM 1077 was transferred to a 15ml sterile centrifuge tube and overlayed with 7.5ml of peripheral blood diluted with 2X volume of sterile isotonic PBS. The tube was centrifuged at 400g for 30-40 minutes and the upper layer containing plasma and platelets was removed without disturbing the mononuclear cell layer at the interface. The layer of mononuclear cells was then transferred to a sterile centrifuge tube and washed twice with PBS. The supernatant was aspirated and resuspended in appropriate volume of complete RPMI‑1640 media supplemented with 10% FBS, 2 mM L-glutamine, 100 IU/ml penicillin and 100 μg/ml streptomycin at 37 °C in a humidified atmosphere containing 5% CO2.

### Co-culture of GluIIß knockout cells with Jurkat E6.1 T cells or PBMCs

Jurkat E6.1 T cells or PBMCs were activated with anti-CD3 (1 μg/ml; eBioscience, ThermoFisher Scientific) and anti-CD28 (1 μg/ml; eBioscience, ThermoFisher Scientific) antibodies for 24 hours. GluIIß knockout, non-target transfected cells were trypsinized and seeded into the well containing activated PBMCs or Jurkat E6.1 T cells at the ratio of 5:1 (immune cells: cancer cells). The co-culturing was continued for 72 hours, afterward culture media was collected and subjected to further tests.

### Assessment of tumor lysing activity of immune cells

After 72 hours of co-culturing of 1.5 x 10^4^ Jurkat E6.1 T cells or PBMCs and 3 x 10^3^ GluIIß knockout A549 cells or non-target transfected cells in 96 well plate, culture media was collected and assesses for tumor lysing activity. Lactate dehydrogenase (LDH) is a cytosolic enzyme present in many different cell types that is released into the cell culture medium upon damage to the plasma membrane. Extracellular LDH was measured by a coupled enzymatic reaction in which LDH catalyzes the conversion of lactate to pyruvate via NAD+ reduction to NADH (CyQUANT LDH Cytotoxicity Assay, ThermoFisher Scientific). Oxidation of NADH by diaphorase resulted in the reduction of a tetrazolium salt (INT) to a red formazan product that was measured spectrophotometrically at 490 nm. The specific lysis was calculated using the following formula, in which the maximum release of LDH activities was obtained from treating the cells with lysis buffer.

$$\lbrack(\mathrm{experimental}\;-\;\mathrm{spontaneous}\;\mathrm{release})/(\mathrm{maximum}\;\mathrm{load}\;-\;\mathrm{spontaneous}\;\mathrm{release})\;\mathrm x\;100(\%)\rbrack$$

### Assessment of proliferation of T cells co-cultured with GluIIß knockout A549 cells

The effect of GluIIß knockout on proliferation of immune cells was investigated by co-culturing GluIIß knockout A549 cells or non-target transfected cells with the Jurkat E6.1 T cells for 24 hours at the ratio of cancer cells to immune cells of 1:5. Afterward, approximately 1 x 10^4^ Jurkat E6.1 T cells were isolated and seed in 96 well plate containing 200 μl of DMEM supplemented with 10% FBS overnight. Cell proliferation was measured with alamarBlue^®^ (Biorad Laboratory, USA) at various time points (0, 12, 24, 48, 72 hours). Twenty microliters of alarmarBlue were added into each well and incubated at 37°C for 4 hours. Fluorescence was then monitored at 560nm excitation wavelength and 590nm emission wavelength.

### Determination of secreted cytokine profile

After the Jurkat E6.1 T cells was activated with anti-CD3 antibodies and anti-CD28 antibodies for 24 hour and co-cultured with GluIIß knockout or non-target transfected A549 cells for another 72 hours, the co-culturing media was collected and subjected to cytokine array measurement using Human Cytokine Antibody Array- Membrane; ab133997, Abcam) following the manufacture’s instruction. In brief, 1 ml of culture media was added onto cytokine array spotted with antibodies specific for different cytokine and incubated for 24 hours at 4 °C. After washing off the unbound fraction, the membrane was incubated with biotinylated detector antibodies and then streptavidin HRP, before subjecting to visualization using chemiluminescent-based detection method. Signal intensity of each cytokine was quantified using “ImageJ software with the Protein Array Analyzer plugin16”. Values from duplicate spots from 2 independent experiments were averaged and plotted as scatter plot and bar graph.

The lists of 42 human cytokines included on the membrane are as follows: ENA-78 (epithelial neutrophil-activating peptide-78), G-CSF (Granulocyte-colony-stimulating factor), GM-CSF (Granulocyte-macrophage colony stimulating factor), GRO alpha (growth-regulated oncogene alpha), GRO beta, GRO gamma, I-309, IL (Interleukin)-1α, IL-1ß, IL-2, IL-3, IL-4, IL-5, IL-6, IL-7, IL-8, IL-10, IL-12 p40/p70, IL-13, IL-15, IFN-gamma, MCP-1 (Monocyte Chemoattractant Protein-1), MCP-2, MCP-3, MCSF (Macrophage Colony-Stimulating Factor), MDC (Macrophage-derived chemokine), MIG (Monokine induced by interferon-gamma), MIP-1 δ (Macrophage inflammatory protein-1 delta), RANTES (Regulated on Activation, Normal T Cell Expressed and Secreted), SCF (Stem cell factor), SDF-1 (Stromal cell-derived factor-1), TARC (Thymus and Activation Regulated Chemokine), TGF (Transforming growth factor)-beta1, TNF (Tumor necrosis factor)-alpha, and TNF-beta, EGF (Epidermal Growth Factor), IGF-1 (Insulin like growth factor I), Angiogenin, Oncostatin M, Thrombopoeitin, VEGF (Vascular endothelial growth factor), PDGF BB (Platelet-Derived Growth Factor-BB) and Leptin.

After the co-culturing, Jurkat E6.1 T cells were separated from GluIIβ KO cells or non-target transfected cells and subjected for RNA isolation and realtime RT-PCR to confirm the results of cytokine array measurement.

### Real-time quantitative RT-PCR

Real-time quantitative RT-PCR was performed to validate RNA sequencing results and cytokine array results. Total RNA was isolated using NucleoSpin RNA Plus (MACHEREY-NAGEL) according to the manufacturer’s instruction. cDNA was synthesis using Tetro cDNA synthesis kit (Bioline, Meridian Bioscience) and subjected to quantitative real-time PCR using primers listed in the Supplementary Table 1. The selected gene targets were amplified with CFX Touch Real-Time PCR Detection (Bio-Rad Laboratories, USA) using 18s rRNA as internal control. The differential gene expression was calculated based on 2^-ΔCт^ value compared to 18s rRNA.

### Statistical analysis

Statistical analyses were performed using SPSS software version 18 (SPSS, Inc., Chicago, IL, USA). The Mann-Whitney U test was used to analyze comparison of means between groups, with *p* < 0.05 considered statistically significant.

## Result

### Transcriptomic expression of mRNAs in cells having GluIIß knockout compared to non-target transfected cells

**Fig. 1:**
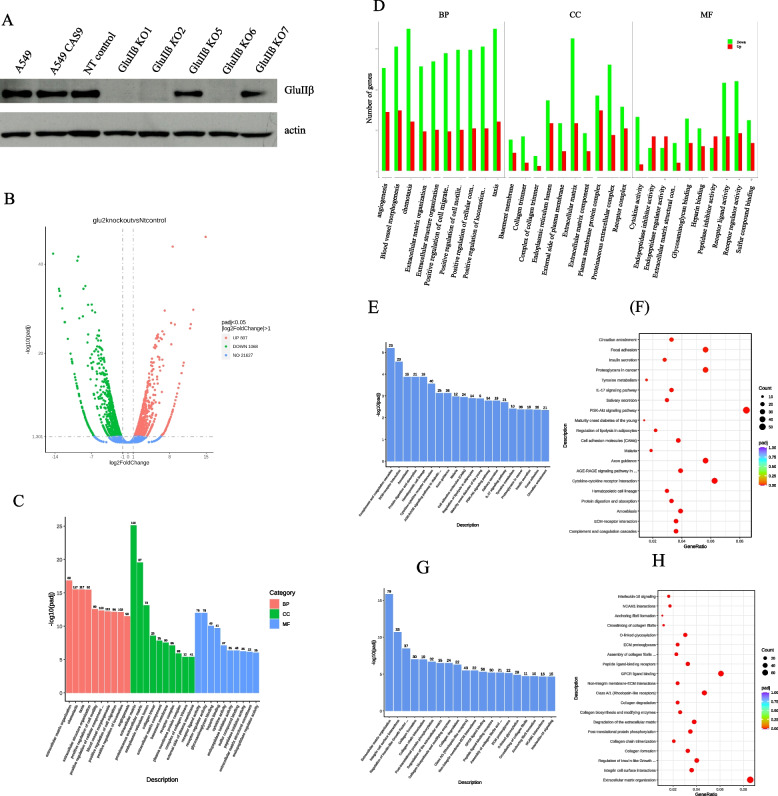
Differential expression, Gene Ontology (GO), KEGG and Reactome analysis compared between GluIIß knockout cells (KO) and non-target transfected cells (control). Western blot analysis showing GluIIß expression levels in GluIIß KO and non-target transfected cells (**A**). Volcano plots (**B**) showing the overall distribution of differentially expressed genes. GO Enrichment analysis histogram showing top 30 significantly (padj <0.05) affected pathways according to major categories of biological processes (BP), cell components (CC), molecular functions (MF) (**C**) and according to categories of up and down expressed genes (**D**) in response to GluIIß knockout. KEGG enrichment analysis histogram (**E**), scatter plot (**F**) and Reactome enrichment analysis histogram (**G**) and scatter plot (**H**) showing pathways significantly (padj <0.05) affected by GluIIß knockout

Verification of GluIIß knockout in the established clones is shown in Fig. 1A, successful suppression of GluIIß expression was observed in knockout clone number 1, 2 and 6 thus they were subjected for further investigations. Comparative gene expression analysis was done between GluIIß knockout clone no.1 and non-target transfected cells. Figure 1B is Volcano plots showing the overall distribution of differentially expressed genes. Out of 23,502 expressed transcripts, a total of 1068 genes showed significant up-regulation and 807 genes showed down-regulation (log 2-fold change > 1, *p<*0.05). List of 26 genes most upregulated and 55 genes down regulated having a log2-fold change of 8.0 or more and less than log2-fold change of -8.0 or less and their roles in cell biology are shown in Tables 1 and 2, respectively.

**Table 1:** List of genes most upregulated in GluIIß knockout A549 cells compared to non-target transfected cells (log 2-fold change value above +8.0)

No.	Gene	Gene ID	Log2(foldchange)	Gene name
1	KRT4	ENSG00000170477	14.9181	keratin 4
2	ADH1C	ENSG00000248144	12.5354	alcohol dehydrogenase 1C (class I), gamma polypeptide
3	ANKS4B	ENSG00000175311	12.056	ankyrin repeat and sterile alpha motif domain containing 4B
4	HGF	ENSG00000019991	11.912	hepatocyte growth factor
5	TPTEP1	ENSG00000100181	10.6873	TPTE pseudogene 1
6	AP000547.3	ENSG00000283633	10.5691	novel transcript
7	GJB1	ENSG00000169562	10.2814	gap junction protein beta 1
8	BPIFB1	ENSG00000125999	9.8638	BPI fold containing family B member 1
9	F7	ENSG00000057593	9.68815	coagulation factor VII
10	H19	ENSG00000130600	9.44138	H19, imprinted maternally expressed transcript
11	AC092336.1	ENSG00000249166	9.29137	novel transcript
12	OR51E2	ENSG00000167332	9.12393	olfactory receptor family 51 subfamily E member 2
13	OR51E1	ENSG00000180785	9.00386	olfactory receptor family 51 subfamily E member 1
14	EDN3	ENSG00000124205	8.95679	endothelin 3
15	PRKCB	ENSG00000166501	8.84155	protein kinase C beta
16	PRR15L	ENSG00000167183	8.63558	proline rich 15 like
17	NTS	ENSG00000133636	8.58469	neurotensin
18	SERPINB11	ENSG00000206072	8.55008	serpin family B member 11 (gene/pseudogene)
19	AC008163.1	ENSG00000233491	8.52041	uncharacterized LOC100128317
20	CES5AP1	ENSG00000215478	8.49012	carboxylesterase 5A pseudogene 1
21	CTNND2	ENSG00000169862	8.36217	catenin delta 2
22	LINC02212	ENSG00000249396	8.31637	long intergenic non-protein coding RNA 2212
23	VIL1	ENSG00000127831	8.28829	villin 1
24	LINC01146	ENSG00000258867	8.22176	long intergenic non-protein coding RNA 1146
25	HOXD-AS2	ENSG00000237380	8.06619	HOXD cluster antisense RNA 2
26	NPY6R	ENSG00000226306	8.02453	neuropeptide Y receptor Y6 (pseudogene)

**Table 2:** List of genes most down regulated in GluIIß knockout A549 cells compared to non-target transfected cells (log 2-fold change value less than -8.0)

**No**	**Gene**	**Gene ID**	**Log2(foldchange)**	**Gene name**
1	DDX3Y	ENSG00000067048	-14.348677	DEAD-box helicase 3 Y-linked
2	RNF182	ENSG00000180537	-13.219258	ring finger protein 182
3	TXLNGY	ENSG00000131002	-13.144863	taxilin gamma pseudogene, Y-linked
4	PNMA8A	ENSG00000182013	-12.989451	PNMA family member 8A
5	EIF1AY	ENSG00000198692	-12.583889	eukaryotic translation initiation factor 1A Y-linked
6	USP9Y	ENSG00000114374	-12.124793	ubiquitin specific peptidase 9 Y-linked
7	ZFY	ENSG00000067646	-11.54406	zinc finger protein Y-linked
8	TTTY15	ENSG00000233864	-11.02679	testis-specific transcript, Y-linked 15
9	UTY	ENSG00000183878	-10.827583	ubiquitously transcribed tetratricopeptide repeat containing, Y-linked
10	ZNF22	ENSG00000165512	-10.738082	zinc finger protein 22
11	FLJ16779	ENSG00000275620	-10.702124	uncharacterized LOC100192386
12	PRKY	ENSG00000099725	-10.665247	protein kinase Y-linked (pseudogene)
13	CDH11	ENSG00000140937	-10.252993	cadherin 11
14	IL6	ENSG00000136244	-10.139271	interleukin 6
15	CSF2	ENSG00000164400	-10.027481	colony stimulating factor 2
16	CES4A	ENSG00000172824	-9.8935981	carboxylesterase 4A
17	HAS2	ENSG00000170961	-9.7877802	hyaluronan synthase 2
18	TNFAIP6	ENSG00000123610	-9.746008	TNF alpha induced protein 6
19	COL5A1	ENSG00000130635	-9.7127701	collagen type V alpha 1 chain
20	TNFSF14	ENSG00000125735	-9.6883611	TNF superfamily member 14
21	FGF5	ENSG00000138675	-9.6883611	fibroblast growth factor 5
22	PTX3	ENSG00000163661	-9.6735821	pentraxin 3
23	RPS4Y1	ENSG00000129824	-9.5360646	ribosomal protein S4 Y-linked 1
24	LINC01139	ENSG00000215808	-9.4315444	long intergenic non-protein coding RNA 1139
25	AL022341.2	ENSG00000262528	-9.3778525	novel transcript, antisense to WDR90
26	RHOD	ENSG00000173156	-9.3409146	ras homolog family member D
27	KIRREL3	ENSG00000149571	-9.3336385	kirre like nephrin family adhesion molecule 3
28	JPH2	ENSG00000149596	-9.0969065	junctophilin 2
29	PDCD1LG2	ENSG00000197646	-9.0969065	programmed cell death 1 ligand 2
30	B4GALNT2	ENSG00000167080	-9.0054488	beta-1,4-N-acetyl-galactosaminyltransferase 2
31	SFRP5	ENSG00000120057	-8.8299628	secreted frizzled related protein 5
32	ZNF738	ENSG00000172687	-8.8299628	zinc finger protein 738
33	FAM224A	ENSG00000233522	-8.8030556	family with sequence similarity 224 member A
34	COL8A1	ENSG00000144810	-8.775637	collagen type VIII alpha 1 chain
35	NLGN4Y	ENSG00000165246	-8.7476871	neuroligin 4 Y-linked
36	KDM5D	ENSG00000012817	-8.639616	lysine demethylase 5D
37	NTSR1	ENSG00000101188	-8.5991885	neurotensin receptor 1
38	AC005307.1	ENSG00000260725	-8.5991885	novel transcript
39	AC108517.1	ENSG00000249111	-8.5352273	novel transcript
40	4-Mar	ENSG00000144583	-8.5260613	membrane associated ring-CH-type finger 4
41	TMSB4Y	ENSG00000154620	-8.5021508	thymosin beta 4 Y-linked
42	CCL5	ENSG00000271503	-8.4682982	C-C motif chemokine ligand 5
43	KLHDC9	ENSG00000162755	-8.4336322	kelch domain containing 9
44	TMEM30B	ENSG00000182107	-8.4336322	transmembrane protein 30B
45	KRT34	ENSG00000131737	-8.4336322	keratin 34
46	GALNT16	ENSG00000100626	-8.3981126	polypeptide N-acetylgalactosaminyltransferase 16
47	NEXN-AS1	ENSG00000235927	-8.324337	NEXN antisense RNA 1
48	KRTAP2-3	ENSG00000212724	-8.324337	keratin associated protein 2-3
49	LINGO2	ENSG00000174482	-8.2465846	leucine rich repeat and Ig domain containing 2
50	AP000695.2	ENSG00000233818	-8.2060783	novel transcript
51	EVI2B	ENSG00000185862	-8.2060783	ecotropic viral integration site 2B
52	MEG3	ENSG00000214548	-8.1609565	maternally expressed 3
53	NUDT16P1	ENSG00000246082	-8.1214854	nudix hydrolase 16 pseudogene 1
54	MMP19	ENSG00000123342	-8.0316218	matrix metallopeptidase 19
55	CDH12	ENSG00000154162	-8.0316218	cadherin 12

### Functional analysis of genes affected by GluIIß knockout

To characterize the functions of gene with significant changes in response to GluIIß knockout, the GO, KEGG and the Reactome pathway analysis was performed using the clusterProfiler [17] software. GO analysis sub-divided pathways into three different categories: cellular component (CC), biological process (BP), and molecular functions (MF). The GO analysis results showed that biological processes down-regulated in response to GluIIß knockout were those involved in angiogenesis, blood vessel morphogenesis, chemotaxis, extracellular matrix (ECM) and structure as well as regulation of cell mobility and communication (Fig. 1C, D). The cellular components by GluIIß knockout belong to the extracellular region, including extracellular matrix component, basement membrane, receptor complex, plasma protein complex, external side of plasma membrane (Fig. 1C, D). The molecular functions of those Differentially Expressed Genes (DEGs) were related to cytokine activity, receptor ligand activity, receptor regulator activity, glycosaminoglycan binding, heparin binding (Fig. 1C, D).

The KEGG pathway analysis revealed that pathways with DEGs were related to the ECM-receptor interaction, cytokine-cytokine receptor interaction, cell adhesion molecules (CAMs), PI3K-Akt signaling pathway and IL-17 signaling pathway (Fig. 1E, F). List of pathways identified by KEGG enrichment analysis significantly upregulated and downregulated by GluIIß knockout are shown in Tables 3 and 4, respectively. The Reactome analysis also identified pathways suppressed by GluIIß knockout involved with ECM organization, ECM proteoglycan, integrin cell surface interaction, non-integrin membrane-ECM interaction and degradation of ECM and IL-10 signaling (Fig. 1G, H). Pathways identified by Reactome enrichment analysis to be significantly upregulated and downregulated by GluIIß knockout are shown in Tables 5 and 6, respectively.

**Table 3:** Pathways in A549 lung adenocarcinoma cells significantly upregulated by GluIIß knockout as identified by the KEGG enrichment analysis

**No.**	**KEGG ID**	**DEG**	**Description**	**padj**	**geneName**	**Count**
1	hsa04918	Up	Thyroid hormone synthesis	0.040819616	ADCY5/GPX2/FXYD2/GPX3/PRKCB/PLCB1/TTR/ADCY9/HSPA5	9
2	hsa04911	Up	Insulin secretion	0.030818158	ADCY5/FXYD2/CACNA1D/PRKCB/KCNJ11/PLCB1/RAB3A/ADCY9/RAPGEF4/ABCC8	10
3	hsa04970	Up	Salivary secretion	0.030818158	ADCY5/MUC5B/FXYD2/CST4/PRKCB/CST2/PLCB1/PRKG1/CST1/ADCY9	10
4	hsa02010	Up	ABC transporters	0.030818158	ABCB5/ABCG2/ABCB1/ABCB4/ABCA2/ABCC4/ABCC8	7
5	hsa04923	Up	Regulation of lipolysis in adipocytes	0.015700313	ADCY5/NPY1R/IRS2/AQP7/PRKG1/PLIN1/ADORA1/ADCY9	8
6	hsa00980	Up	Metabolism of xenobiotics by cytochrome P450	0.013693988	ADH1C/ADH6/UGT1A1/CYP1A1/ALDH3A1/UGT1A6/AKR1C1/EPHX1/CYP2S1	9
7	hsa00360	Up	Phenylalanine metabolism	0.010209358	DDC/HPD/ALDH3A1/TAT/MAOA	5
8	hsa00910	Up	Nitrogen metabolism	0.004642571	CPS1/CA8/CA2/GLUL/CA13	5
9	hsa00830	Up	Retinol metabolism	0.002769283	ADH1C/ADH6/UGT1A1/CYP1A1/CYP26B1/UGT1A6/SDR16C5/RDH10/CYP2S1/ALDH1A1	10
10	hsa04976	Up	Bile secretion	0.002769283	ADCY5/FXYD2/CA2/NR1H4/ABCG2/SLC51B/ABCB1/ABCB4/ADCY9/EPHX1/ABCC4	11
11	hsa04950	Up	Maturity onset diabetes of the young	0.001518883	HNF4A/HNF1A/RFX6/FOXA3/HNF4G/BHLHA15/HHEX	7
12	hsa00350	Up	Tyrosine metabolism	0.001518883	ADH1C/HGD/DDC/ADH6/HPD/ALDH3A1/TAT/MAOA	8
13	hsa04610	Up	Complement and coagulation cascades	0.000147849	FGB/F5/FGG/FGA/F7/F2/PROC/F2RL2/CPB2/F13B/VTN/C5/TFPI/KNG1	14

**Table 4:** Pathways in A549 lung adenocarcinoma cells significantly downregulated by GluIIß knockout as identified by the KEGG enrichment analysis

**No.**	**KEGG ID**	**DEG**	**Description**	**padj**	**geneName**	**Count**
1	hsa04060	Down	Cytokine-cytokine receptor interaction	1.64E-06	IL11/CXCL5/CXCL1/IL31RA/CXCL8/IL6/CSF3/CSF2/TNFSF14/CXCL3/CCL20/IL32/CXCL2/IL33/IL7R/TNFRSF19/BMP2/INHBA/CD70/IL1B/TNFRSF25/TNFRSF9/CCL2/NGF/IL1A/TGFB2/CRLF2/CCL26/IL24/PRLR/CSF2RA/PPBP/TGFB1	33
2	hsa04640	Down	Hematopoietic cell lineage	1.64E-06	ANPEP/IL11/IL6/CSF3/CSF2/ITGB3/IL7R/ITGA4/IL1B/IL1A/ITGA2/CD38/ITGA5/CD14/CSF2RA/CD55/CD44	17
3	hsa04512	Down	ECM-receptor interaction	4.11E-06	TNC/ITGB3/COL4A1/COL4A6/ITGA4/COL6A2/LAMC2/LAMA4/COL6A1/COL4A2/ITGA2/COL4A5/THBS1/ITGA5/SDC4/ITGB6/CD44/COL1A1	18
4	hsa05146	Down	Amoebiasis	6.04E-06	CXCL1/TLR4/CXCL8/IL6/CSF2/SERPINB3/COL4A1/COL4A6/SERPINB4/LAMC2/IL1B/LAMA4/PIK3CD/TGFB2/COL4A2/COL4A5/CD14/RAB7B/TGFB1/COL1A1	20
5	hsa04657	Down	IL-17 signaling pathway	1.29E-05	S100A9/CXCL5/CXCL1/CXCL8/IL6/CSF3/CSF2/TNFAIP3/CXCL3/CCL20/CXCL2/PTGS2/MMP1/LCN2/IL1B/CCL2/S100A8/FOSL1/JUN	19
6	hsa05323	Down	Rheumatoid arthritis	2.03E-05	IL11/CXCL5/CXCL1/TLR4/CXCL8/IL6/CSF2/CCL20/MMP1/IL1B/CCL2/IL1A/TGFB2/ICAM1/JUN/TGFB1	16
7	hsa05144	Down	Malaria	2.79E-05	TLR4/CXCL8/IL6/CSF3/IL1B/CCL2/TGFB2/ICAM1/THBS1/LRP1/TGFB1	11
8	hsa04668	Down	TNF signaling pathway	9.53E-05	CXCL5/CXCL1/IL6/CSF2/TNFAIP3/CXCL3/CCL20/CXCL2/SOCS3/PTGS2/IL1B/PIK3CD/CCL2/VEGFC/ICAM1/CREB5/JUN/TRAF1/RPS6KA5/BCL3	20
9	hsa04933	Down	AGE-RAGE signaling pathway in diabetic complications	0.000106817	SERPINE1/CXCL8/IL6/COL4A1/COL4A6/IL1B/STAT5A/MMP2/PIK3CD/CCL2/IL1A/TGFB2/COL4A2/COL4A5/VEGFC/ICAM1/JUN/TGFB1/COL1A1	19
10	hsa05205	Down	Proteoglycans in cancer	0.000126055	PLAU/TLR4/ANK1/ITGB3/WNT5A/CAV1/LUM/MMP2/PIK3CD/HOXD10/TGFB2/ITGA2/EGFR/THBS1/ITGA5/PLAUR/SDC4/TWIST1/FZD2/ITPR2/HBEGF/CAMK2A/CAMK2B/FGF2/CAV2/ITPR1/TGFB1/CD44	28
11	hsa04151	Down	PI3K-Akt signaling pathway	0.00020725	TNC/GNG2/TLR4/IL6/CSF3/FGF5/ITGB3/IL7R/COL4A1/COL4A6/ITGA4/EFNA1/COL6A2/LAMC2/LAMA4/PPP2R2B/COL6A1/SGK1/F2R/PIK3CD/NGF/COL4A2/ITGA2/COL4A5/VEGFC/FGF1/EGFR/CREB5/THBS1/ITGA5/PRLR/NTRK2/PDGFRA/GNGT2/ITGB6/FGF2/EFNA3/COL1A1	38
12	hsa04514	Down	Cell adhesion molecules (CAMs)	0.000897575	CDH4/CDH2/PDCD1LG2/VCAN/ITGA4/NLGN4Y/NEGR1/NRXN3/ESAM/CD274/ICAM1/PTPRF/CADM1/SDC4/NECTIN3/NLGN2/NFASC	17
13	hsa04360	Down	Axon guidance	0.000928331	UNC5D/EPHA5/SLIT3/EPHB2/RHOD/EFNA1/WNT5A/SEMA3F/EPHA4/NTN4/PIK3CD/EFNB2/ABLIM3/SEMA3A/SLIT2/EFNB3/NGEF/NRP1/PLXNB3/BOC/CAMK2A/CAMK2B/EFNA3/SEMA7A	24
14	hsa04926	Down	Relaxin signaling pathway	0.002116087	GNG2/NOS1/COL4A1/COL4A6/MMP1/MMP2/PIK3CD/SHC4/COL4A2/SHC3/COL4A5/VEGFC/EGFR/CREB5/JUN/ACTA2/GNGT2/TGFB1/COL1A1	19
15	hsa04510	Down	Focal adhesion	0.002118851	TNC/ITGB3/COL4A1/COL4A6/ITGA4/COL6A2/CAV1/LAMC2/LAMA4/COL6A1/PIK3CD/SHC4/COL4A2/SHC3/ITGA2/COL4A5/VEGFC/EGFR/THBS1/ITGA5/JUN/PDGFRA/ITGB6/CAV2/COL1A1	25
16	hsa05206	Down	MicroRNAs in cancer	0.003564802	TNC/PLAU/ZEB2/ITGB3/PTGS2/SOX4/FSCN1/HOXD10/TGFB2/SHC4/HMGA2/TPM1/TP63/EGFR/THBS1/ITGA5/BMF/RPS6KA5/PDGFRA/ZFPM2/EFNA3/CD44	22
17	hsa04974	Down	Protein digestion and absorption	0.006872919	COL5A1/COL13A1/COL4A1/COL4A6/KCNN4/COL6A2/COL7A1/COL6A1/COL4A2/COL4A5/COL17A1/COL1A1	12
18	hsa04630	Down	JAK-STAT signaling pathway	0.007649023	IL11/IL6/CSF3/CSF2/IL7R/SOCS3/STAT5A/PIK3CD/SOCS2/CRLF2/EGFR/IL24/AOX1/PRLR/CSF2RA/PDGFRA/IL22RA2	17
19	hsa05134	Down	Legionellosis	0.008413694	C3/CXCL1/TLR4/CXCL8/IL6/CXCL3/CXCL2/IL1B/HSPA2/CD14	10
20	hsa05132	Down	Salmonella infection	0.010368895	CXCL1/TLR4/CXCL8/IL6/CSF2/CXCL3/CXCL2/IL1B/IL1A/RILP/CD14/JUN/RAB7B	13
21	hsa04621	Down	NOD-like receptor signaling pathway	0.010379084	CXCL1/TLR4/CXCL8/IFI16/IL6/TNFAIP3/CXCL3/CXCL2/ANTXR1/IL1B/CTSB/CCL2/ANTXR2/NLRP1/GBP1/JUN/GBP2/ITPR2/ITPR1	19
22	hsa05200	Down	Pathways in cancer	0.01238625	GNG2/CTNNA2/CXCL8/IL6/FGF5/IL7R/BMP2/COL4A1/COL4A6/PTGS2/RUNX1/MMP1/WNT5A/LAMC2/STAT5A/LAMA4/F2R/MMP2/PIK3CD/TGFB2/COL4A2/PLEKHG5/ITGA2/GLI2/COL4A5/VEGFC/FGF1/RASGRP1/EGFR/BIRC7/JUN/FZD2/TRAF1/AXIN2/CSF2RA/RPS6KA5/PDGFRA/GNGT2/PPARG/NKX3-1/CAMK2A/CAMK2B/FGF2/ETS1/TGFB1	45
23	hsa05133	Down	Pertussis	0.018621623	CXCL5/C3/TLR4/CXCL8/IL6/IL1B/IL1A/C1R/ITGA5/CD14/JUN	11
24	hsa05143	Down	African trypanosomiasis	0.028554735	IL6/IL1B/LAMA4/F2RL1/APOL1/ICAM1	6
25	hsa04145	Down	Phagosome	0.028742909	C3/OLR1/TLR4/NOS1/ITGB3/SFTPD/CTSS/RILP/ITGA2/TUBA1A/C1R/THBS1/ITGA5/CD14/RAB7B	15
26	hsa04020	Down	Calcium signaling pathway	0.029706359	NOS1/DRD1/NTSR1/F2R/ADRB2/HRH1/CD38/ADRB1/EGFR/RYR3/PTAFR/PDGFRA/ITPR2/SPHK1/CAMK2A/CAMK2B/GRIN2C/ITPR1	18
27	hsa05410	Down	Hypertrophic cardiomyopathy (HCM)	0.030147705	IL6/ITGB3/ITGA4/TGFB2/ITGA2/SGCD/TPM1/DMD/ITGA5/ITGB6/TGFB1	11
28	hsa05140	Down	Leishmaniasis	0.031475877	C3/TLR4/ITGA4/PTGS2/IL1B/IL1A/TGFB2/JUN/TGFB1	9
29	hsa04064	Down	NF-kappa B signaling pathway	0.032287711	BCL2A1/PLAU/TLR4/CXCL8/TNFAIP3/TNFSF14/CXCL2/PTGS2/IL1B/ICAM1/CD14/TRAF1	12
30	hsa05414	Down	Dilated cardiomyopathy (DCM)	0.038511016	ITGB3/ITGA4/TGFB2/ITGA2/SGCD/TPM1/ADRB1/DMD/ITGA5/ITGB6/TGFB1	11
31	hsa05412	Down	Arrhythmogenic right ventricular cardiomyopathy (ARVC)	0.040130882	CTNNA2/CDH2/ITGB3/ITGA4/ITGA2/SGCD/DMD/ITGA5/GJA1/ITGB6	10
32	hsa05142	Down	Chagas disease (American trypanosomiasis)	0.040130882	C3/SERPINE1/TLR4/CXCL8/IL6/IL1B/PPP2R2B/PIK3CD/CCL2/TGFB2/JUN/TGFB1	12
33	hsa04060	Down	Cytokine-cytokine receptor interaction	1.64E-06	IL11/CXCL5/CXCL1/IL31RA/CXCL8/IL6/CSF3/CSF2/TNFSF14/CXCL3/CCL20/IL32/CXCL2/IL33/IL7R/TNFRSF19/BMP2/INHBA/CD70/IL1B/TNFRSF25/TNFRSF9/CCL2/NGF/IL1A/TGFB2/CRLF2/CCL26/IL24/PRLR/CSF2RA/PPBP/TGFB1	33

**Table 5:** Pathways in A549 lung adenocarcinoma cells significantly upregulated by GluIIß knockout as identified by the Reactome enrichment analysis

No.	Reactome ID	Description	padj	geneName	Count
1	R-HSA-211945	Phase I - Functionalization of compounds	0.000129392	ADH1C/CYP4F11/CYP4F3/ADH6/CYP4F12/NR1H4/CYP1A1/CYP26B1/ ALDH3A1/EPHX1/CES1/CYP2S1/CYP4F2/MAOA/CYP39A1/TBXAS1/ALDH1A1	17
2	R-HSA-211859	Biological oxidations	0.000129392	ADH1C/CYP4F11/CYP4F3/SULT1A1/ADH6/UGT1A1/ACY3/CYP4F12/NR1H4/ CYP1A1/CYP26B1/ALDH3A1/UGT1A6/OPLAH/ MAT1A/EPHX1/UGT2B10/CES1/SLC26A1/CYP2S1/CYP4F2/MAOA/ CYP39A1/TBXAS1/ALDH1A1	25
3	R-HSA-140877	Formation of Fibrin Clot (Clotting Cascade)	0.000197042	FGB/F5/FGG/FGA/F7/F2/PROC/F13B/TFPI/KNG1	10
4	R-HSA-140875	Common Pathway of Fibrin Clot Formation	0.00465798	FGB/F5/FGG/FGA/F2/PROC/F13B	7
5	R-HSA-381426	Regulation of Insulin-like Growth Factor (IGF) transport and uptake by Insulin-like Growth Factor Binding Proteins (IGFBPs)	0.00465798	F5/FGG/FGA/F2/ITIH2/CP/SERPINA1/SPP1/SERPINA10/PROC/DNAJC3/IGFBP5/ VWA1/AHSG/MGAT4A/KNG1	16
6	R-HSA-8957275	Post-translational protein phosphorylation	0.004959453	F5/FGG/FGA/ITIH2/CP/SERPINA1/SPP1/SERPINA10/PROC/DNAJC3/ IGFBP5/VWA1/AHSG/MGAT4A/KNG1	15
7	R-HSA-211897	Cytochrome P450 - arranged by substrate type	0.008079457	CYP4F11/CYP4F3/CYP4F12/NR1H4/CYP1A1/CYP26B1/ CYP2S1/CYP4F2/CYP39A1/TBXAS1	10
8	R-HSA-6788656	Histidine, lysine, phenylalanine, tyrosine, proline and tryptophan catabolism	0.008079457	HGD/HPD/ASRGL1/TAT/PRODH2/ACMSD/SLC7A5/HYKK/SLC3A2	9
9	R-HSA-977068	Termination of O-glycan biosynthesis	0.008079457	MUC5B/MUC13/MUC5AC/ST6GALNAC2/ST3GAL1/ST6GAL1	6
10	R-HSA-76009	Platelet Aggregation (Plug Formation)	0.010006115	FGB/FGG/FGA/F2/ADRA2A/SRC/SYK/RAPGEF4	8
11	R-HSA-383280	Nuclear Receptor transcription pathway	0.014152284	HNF4A/PGR/NR3C2/NR1H4/ESRRG/RORC/NR0B1/HNF4G/PPARA	9
12	R-HSA-5365859	RA biosynthesis pathway	0.016767081	ADH1C/CYP26B1/CRABP1/SDR16C5/RDH10/ALDH1A1	6
13	R-HSA-375165	NCAM signaling for neurite out-growth	0.020245365	CACNA1D/CACNB4/ST8SIA4/COL4A3/SPTBN4/CACNA1H/SPTBN2/COL5A3/COL4A4/SRC	10
14	R-HSA-373076	Class A/1 (Rhodopsin-like receptors)	0.023104286	NTS/P2RY6/NPY1R/F2/EDN3/AGT/LPAR6/GPR37/NPY5R/MTNR1A/S1PR5/F2RL2/ AGTR1/ADORA1/SSTR5/ADRA2A/C5/PLPPR1/KNG1	19
15	R-HSA-2219530	Constitutive Signaling by Aberrant PI3K in Cancer	0.024275153	HGF/NRG4/FGFR3/EGF/FGFR4/ERBB3/IRS2/SRC/FGFR2	9
16	R-HSA-202733	Cell surface interactions at the vascular wall	0.028423039	SLC7A7/TSPAN7/CEACAM1/F2/CEACAM6/PROC/SLC7A5/ SLC3A2/SLC7A11/SRC/SELL/GRB7/EPCAM/CEACAM5	14
17	R-HSA-913709	O-linked glycosylation of mucins	0.029117436	MUC5B/MUC13/B3GNT3/MUC5AC/B3GNT9/GALNT4/ST6GALNAC2/ST3GAL1/ST6GAL1	9
18	R-HSA-71182	Phenylalanine and tyrosine catabolism	0.03745781	HGD/HPD/ASRGL1/TAT	4
19	R-HSA-210745	Regulation of gene expression in beta cells	0.03745781	HNF4A/HNF1A/RFX6/FOXA3/HNF4G	5
20	R-HSA-500792	GPCR ligand binding	0.03745781	NTS/P2RY6/NPY1R/F2/EDN3/AGT/LPAR6/GPR37/NPY5R/MTNR1A/ S1PR5/F2RL2/CALCRL/AGTR1/CALCR/FZD5/ADORA1/SSTR5/ADRA2A/ C5/PLPPR1/ADM2/KNG1/WNT4	24
21	R-HSA-112316	Neuronal System	0.042644478	ADCY5/TSPAN7/PRKCB/LIN7A/GLUL/KCNJ11/CACNB4/RPS6KA2/ HCN4/CPLX1/NEFL/PLCB1/SLITRK6/EPB41L5/PANX2/KCNJ3/KCNS3/ RAB3A/ADCY9/NCALD/CACNA2D1/GRIK4/MAOA/ABCC8/KCNV1/SYT7	26
22	R-HSA-5173105	O-linked glycosylation	0.046099181	MUC5B/MUC13/B3GNT3/ADAMTS1/MUC5AC/B3GNT9/THSD7A/ GALNT4/ADAMTS17/ST6GALNAC2/ST3GAL1/ST6GAL1	12
23	R-HSA-354192	Integrin alphaIIb beta3 signaling	0.046099181	FGB/FGG/FGA/SRC/SYK/RAPGEF4	6
24	R-HSA-5579029	Metabolic disorders of biological oxidation enzymes	0.046099181	UGT1A1/CYP26B1/OPLAH/MAT1A/MAOA/TBXAS1	6
25	R-HSA-9006921	Integrin signaling	0.046099181	FGB/FGG/FGA/SRC/SYK/RAPGEF4	6
26	R-HSA-76002	Platelet activation, signaling and aggregation	0.047697187	HGF/FGB/F5/FGG/FGA/ARRB1/APOH/F2/PRKCB/EGF/SERPINA1/F2RL2/ CFD/VAV3/ADRA2A/SRC/SYK/AHSG/ABCC4/RAPGEF4/KNG1/HSPA5	22
27	R-HSA-419037	NCAM1 interactions	0.047697187	CACNA1D/CACNB4/ST8SIA4/COL4A3/CACNA1H/COL5A3/COL4A4	7
28	R-HSA-211945	Phase I - Functionalization of compounds	0.000129392	ADH1C/CYP4F11/CYP4F3/ADH6/CYP4F12/NR1H4/CYP1A1/CYP26B1/ ALDH3A1/EPHX1/CES1/CYP2S1/CYP4F2/MAOA/CYP39A1/TBXAS1/ALDH1A1	17

**Table 6:** Pathways in A549 lung adenocarcinoma cells significantly downregulated by GluIIß knockout as identified by the Reactome enrichmenr analysis

**No.**	**Reactome ID**	**Description**	**padj**	**geneName**	**Count**
1	R-HSA-1474244	Extracellular matrix organization	1.90E-18	COL5A1/ADAM19/TNC/SERPINE1/NID2/LTBP2/ITGB3/COL13A1/FBN1/FBN2/VCAN/ BMP2/COL4A1/COL4A6/ITGA4/COL8A1/COL20A1/MMP1/LOXL2/COL6A2/MMP10/ LAMC2/EFEMP1/COL7A1/LAMA4/LUM/TIMP2/MMP19/CTSB/COL16A1/COL6A1/ MMP2/NTN4/TGFB2/TLL1/COL4A2/CTSS/ADAMTS16/ITGA2/MMP17/COL4A5/P4HA3/ ICAM1/COL17A1/COLGALT2/DMD/THBS1/ITGA5/SDC4/MFAP5/ADAMTS14/PLOD2/ ADAM12/ITGB6/FBLN2/FGF2/TGFB1/COL28A1/CD44/COL1A1	60
2	R-HSA-216083	Integrin cell surface interactions	4.70E-09	COL5A1/TNC/ITGB3/COL13A1/FBN1/COL4A1/COL4A6/ITGA4/COL8A1/COL6A2/ COL7A1/LUM/COL16A1/COL6A1/COL4A2/ITGA2/COL4A5/ICAM1/THBS1/ITGA5/ ITGB6/CD44/COL1A1	23
3	R-HSA-1474290	Collagen formation	6.76E-09	COL5A1/COL13A1/COL4A1/COL4A6/COL8A1/COL20A1/LOXL2/COL6A2/LAMC2/ COL7A1/CTSB/COL16A1/COL6A1/TLL1/COL4A2/CTSS/COL4A5/P4HA3/COL17A1/ COLGALT2/ADAMTS14/PLOD2/COL28A1/COL1A1	24
4	R-HSA-1650814	Collagen biosynthesis and modifying enzymes	1.56E-08	COL5A1/COL13A1/COL4A1/COL4A6/COL8A1/COL20A1/COL6A2/COL7A1/COL16A1/ COL6A1/TLL1/COL4A2/COL4A5/P4HA3/COL17A1/COLGALT2/ADAMTS14/PLOD2/ COL28A1/COL1A1	20
5	R-HSA-1474228	Degradation of the extracellular matrix	3.48E-08	COL5A1/COL13A1/FBN1/FBN2/COL4A1/COL4A6/COL8A1/MMP1/COL6A2/MMP10/ LAMC2/COL7A1/TIMP2/MMP19/CTSB/COL16A1/COL6A1/MMP2/TLL1/COL4A2/ CTSS/ADAMTS16/MMP17/COL4A5/COL17A1/CD44/COL1A1	27
6	R-HSA-1442490	Collagen degradation	6.34E-08	COL5A1/COL13A1/COL4A1/COL4A6/COL8A1/MMP1/COL6A2/MMP10/COL7A1/ MMP19/CTSB/COL16A1/COL6A1/MMP2/COL4A2/COL4A5/COL17A1/COL1A1	18
7	R-HSA-8948216	Collagen chain trimerization	7.58E-08	COL5A1/COL13A1/COL4A1/COL4A6/COL8A1/COL20A1/COL6A2/COL7A1/ COL16A1/COL6A1/COL4A2/COL4A5/COL17A1/COL28A1/COL1A1	15
8	R-HSA-6783783	Interleukin-10 signaling	1.88E-07	CXCL1/CXCL8/IL6/CSF3/CSF2/CCL20/CXCL2/PTGS2/CCL5/IL1B/CCL2/IL1A/ ICAM1/PTAFR	14
9	R-HSA-3000178	ECM proteoglycans	5.85E-07	COL5A1/TNC/SERPINE1/ITGB3/VCAN/COL4A1/COL4A6/COL6A2/LAMA4/LUM/ COL6A1/TGFB2/COL4A2/ITGA2/COL4A5/ITGB6/TGFB1/COL1A1	18
10	R-HSA-3000171	Non-integrin membrane-ECM interactions	1.54E-06	COL5A1/TNC/ITGB3/COL4A1/COL4A6/LAMC2/LAMA4/NTN4/COL4A2/ITGA2/ COL4A5/DMD/THBS1/SDC4/FGF2/TGFB1/COL1A1	17
11	R-HSA-2022090	Assembly of collagen fibrils and other multimeric structures	5.46E-06	COL5A1/COL4A1/COL4A6/COL8A1/LOXL2/COL6A2/LAMC2/COL7A1/CTSB/ COL6A1/TLL1/COL4A2/CTSS/COL4A5/COL17A1/COL1A1	16
12	R-HSA-1566948	Elastic fibre formation	1.45E-05	LTBP2/ITGB3/FBN1/FBN2/BMP2/LOXL2/EFEMP1/TGFB2/ITGA5/MFAP5/ITGB6/ FBLN2/TGFB1	13
13	R-HSA-381426	Regulation of Insulin-like Growth Factor (IGF) transport and uptake by Insulin-like Growth Factor Binding Proteins (IGFBPs)	3.58E-05	TNC/IGFBP7/C3/EVA1A/IL6/FSTL1/CDH2/PRKCSH/FBN1/VCAN/MFGE8/MMP1/ PAPPA/IGFBP6/MMP2/APOL1/VGF/CYR61/TMEM132A/NOTUM/MSLN	21
14	R-HSA-2214320	Anchoring fibril formation	4.10E-05	COL4A1/COL4A6/LAMC2/COL7A1/TLL1/COL4A2/COL4A5/COL1A1	8
15	R-HSA-2129379	Molecules associated with elastic fibres	7.17E-05	LTBP2/ITGB3/FBN1/FBN2/BMP2/EFEMP1/TGFB2/MFAP5/ITGB6/FBLN2/TGFB1	11
16	R-HSA-6785807	Interleukin-4 and 13 signaling	0.000406901	CXCL8/IL6/SOX2/SOCS3/PTGS2/SAA1/MMP1/LCN2/IL1B/FSCN1/MMP2/CCL2/ IL1A/ICAM1/TWIST1/MUC1/FGF2/TGFB1	18
17	R-HSA-3000170	Syndecan interactions	0.000797158	COL5A1/TNC/ITGB3/ITGA2/THBS1/SDC4/FGF2/TGFB1/COL1A1	9
18	R-HSA-375276	Peptide ligand-binding receptors	0.000892778	CXCL5/C3/CXCL1/CXCL8/CXCL3/CCL20/CXCL2/NPY4R2/LOC105379861/ SAA1/EDN2/NTSR1/CCL5/F2RL1/F2R/NPY4R/PPBP/C5AR2	18
19	R-HSA-500792	GPCR ligand binding	0.001395839	CXCL5/C3/GNG2/CXCL1/CXCL8/DRD1/CXCL3/CCL20/CXCL2/NPY4R2/ LOC105379861/SAA1/EDN2/NTSR1/WNT5A/CCL5/UCN2/F2RL1/F2R/ NPY4R/ADRB2/HRH1/GIP/ADGRE1/ADRB1/FZD2/PTAFR/CD55/PPBP/C5AR2/GNGT2/S1PR3	32
20	R-HSA-380108	Chemokine receptors bind chemokines	0.001395839	CXCL5/CXCL1/CXCL8/CXCL3/CCL20/CXCL2/CCL5/PPBP	8
21	R-HSA-8957275	Post-translational protein phosphorylation	0.001395839	TNC/IGFBP7/C3/EVA1A/IL6/FSTL1/CDH2/PRKCSH/FBN1/VCAN/MFGE8/ APOL1/VGF/CYR61/TMEM132A/NOTUM/MSLN	17
22	R-HSA-3000157	Laminin interactions	0.001599807	NID2/COL4A1/COL4A6/LAMC2/COL7A1/LAMA4/COL4A2/ITGA2/COL4A5	9
23	R-HSA-2243919	Crosslinking of collagen fibrils	0.001704251	COL4A1/COL4A6/LOXL2/TLL1/COL4A2/COL4A5/COL1A1	7
24	R-HSA-373076	Class A/1 (Rhodopsin-like receptors)	0.001803729	CXCL5/C3/CXCL1/CXCL8/DRD1/CXCL3/CCL20/CXCL2/NPY4R2/LOC105379861/ SAA1/EDN2/NTSR1/CCL5/F2RL1/F2R/NPY4R/ADRB2/HRH1/ADRB1/PTAFR/PPBP /C5AR2/S1PR3	24
25	R-HSA-2173782	Binding and Uptake of Ligands by Scavenger Receptors	0.002478535	JCHAIN/COL4A1/SAA1/SCARA5/APOL1/COL4A2/LRP1/SCARF1/COL1A1	9
26	R-HSA-5173105	O-linked glycosylation	0.003109464	GALNT5/GALNT16/GALNT9/ADAMTSL4/ADAMTSL1/ADAMTS16/SPON2/GALNT6/ ADAMTS12/ADAMTS6/THBS1/B3GNT7/B3GNT5/ADAMTS14/MUC3A/MUC1	16
27	R-HSA-449147	Signaling by Interleukins	0.003267263	IL11/CXCL1/IL31RA/CXCL8/IL6/CSF3/CSF2/SOX2/CCL20/IL32/CXCL2/IL33/IL7R/ SOCS3/SOD2/PTGS2/SAA1/EBI3/MMP1/LCN2/IRAK2/CCL5/IL1B/STAT5A/FSCN1/ MMP2/PIK3CD/CCL2/IL1A/SOCS2/CRLF2/ICAM1/IL24/JUN/TWIST1/CSF2RA/ PTAFR/RPS6KA5/MUC1/FGF2/IL22RA2/TGFB1	42
28	R-HSA-419037	NCAM1 interactions	0.007391944	COL5A1/COL4A1/COL6A2/GFRA1/COL6A1/COL4A2/GFRA2/COL4A5/GDNF	9
29	R-HSA-75205	Dissolution of Fibrin Clot	0.007391944	SERPINE1/PLAU/PLAT/SERPINE2/PLAUR	5
30	R-HSA-5083635	Defective B3GALTL causes Peters-plus syndrome (PpS)	0.007829636	ADAMTSL4/ADAMTSL1/ADAMTS16/SPON2/ADAMTS12/ADAMTS6/THBS1/ADAMTS14	8
31	R-HSA-418990	Adherens junctions interactions	0.009382596	CDH11/CDH4/CDH2/CDH12/CDH8/CDH6/CADM1/NECTIN3	8
32	R-HSA-5173214	O-glycosylation of TSR domain-containing proteins	0.009382596	ADAMTSL4/ADAMTSL1/ADAMTS16/SPON2/ADAMTS12/ADAMTS6/THBS1/ADAMTS14	8
33	R-HSA-186797	Signaling by PDGF	0.021385068	COL5A1/PLAT/COL4A1/COL6A2/STAT5A/COL6A1/COL4A2/COL4A5/THBS1/PDGFRA	10
34	R-HSA-3906995	Diseases associated with O-glycosylation of proteins	0.021385068	ADAMTSL4/ADAMTSL1/ADAMTS16/SPON2/ADAMTS12/ADAMTS6/THBS1/ ADAMTS14/MUC3A/MUC1	10
35	R-HSA-375165	NCAM signaling for neurite out-growth	0.041925906	COL5A1/COL4A1/COL6A2/GFRA1/COL6A1/COL4A2/GFRA2/COL4A5/RPS6KA5/GDNF	10
36	R-HSA-3928665	EPH-ephrin mediated repulsion of cells	0.041925906	EPHA5/EPHB2/EFNA1/CLTCL1/EPHA4/MMP2/EFNB2/EFNB3/EFNA3	9
37	R-HSA-5686938	Regulation of TLR by endogenous ligand	0.041925906	S100A9/TLR4/SFTPD/S100A8/CD14	5
38	R-HSA-2980736	Peptide hormone metabolism	0.041925906	ANPEP/PCSK1/INHBA/PAX6/CGB8/ENPEP/GIP/CGB5/KIF5A/ACHE/MYRIP	11

### Verification of expression levels of CAMs in GluIIβ knockout cells by quantitative real-time PCR and cell morphology

**Fig. 2:**
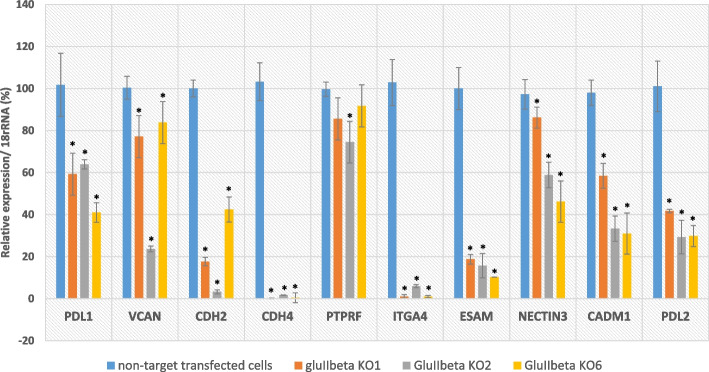
Quantitative RT-PCR validation of significant changes demonstrated by NGS data. 9 genes encoded for CAMs were chosen for verification and 8 genes were confirmed to be significantly decreased in all 3 GluIIß knockout clones (PD-L1, VCAN, CDH2, CDH4, ITGA4, ESAM, NECTIN3, CADM1) while PTPRF was significantly decreased only in 1 out of 3 clones compared to non-target transfected cells. Further investigation on the effect of GluIIß knockout on PD-L2 also demonstrated the significant impact on the down regulation of PD-L2

**Fig. 3:**
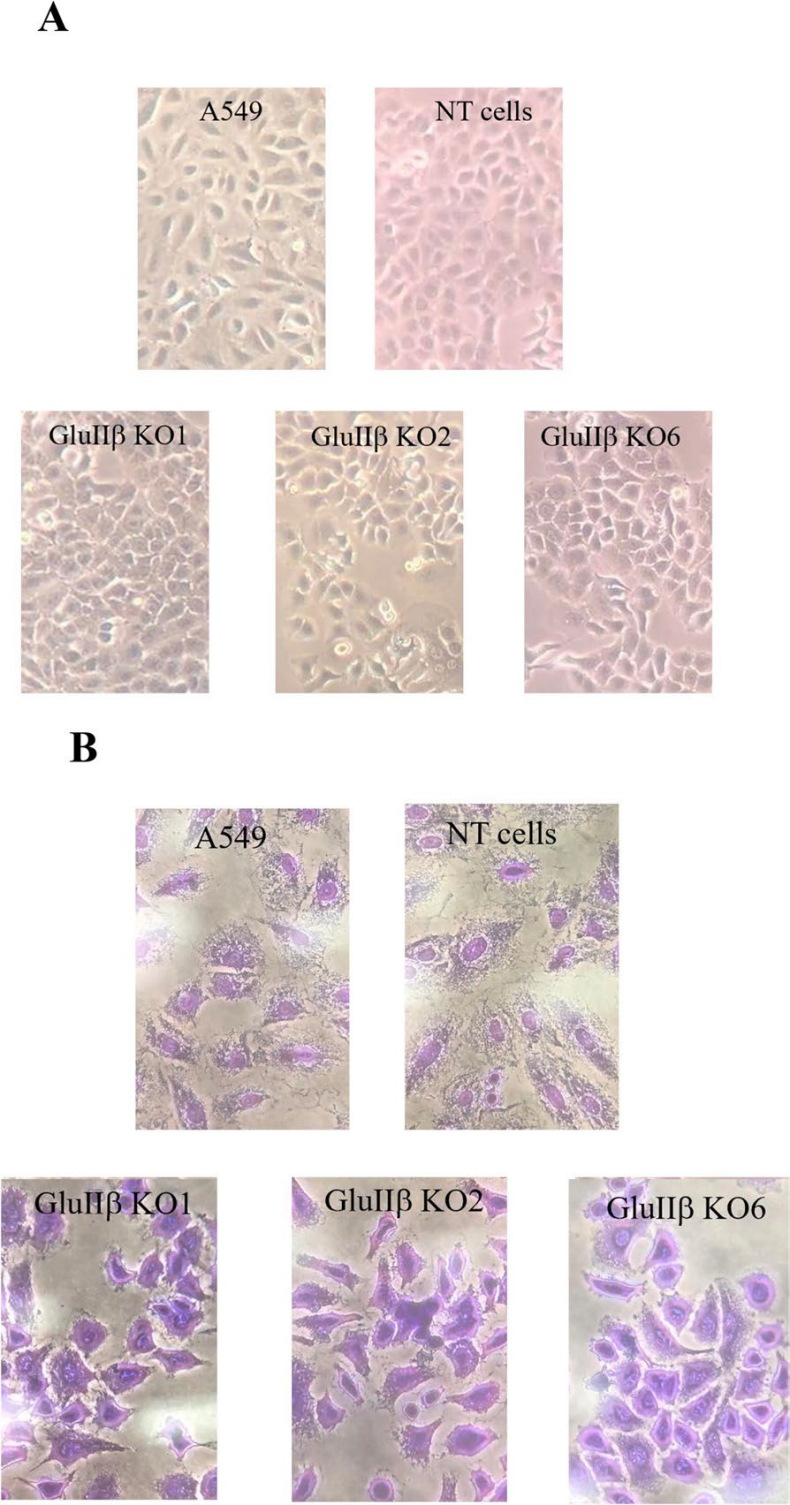
Phase-contrast microscopy (**A**) and crystal violet staining (**B**) images at 40x magnification of GluIIß KO cells (KO1, KO2, KO6) compared to parental (A549) and non-target transfected cells (NT)

Real-time quantitative RT-PCR was performed to validate expression levels of 9 genes encoded for cell adhesion molecules identified by KEGG to be significantly impacted by GluIIβ knockout. These 9 selected genes were as followed: CDH4 (cadherin 4), CDH2 (cadherin 2), VCAN (versican), ITGA4 (integrin subunit alpha 4), ESAM (endothelial cell-selective adhesion molecule), CD274 (program death-ligand-1 (PD-L1)), PTPRF (Protein Tyrosine Phosphatase Receptor Type F), CADM1 (Cell Adhesion Molecule 1), and NECTIN3 (Nectin Cell Adhesion Molecule 3). Since PD-L2 is a second ligand for PD-1 and their interaction dramatically inhibited T cell receptor (TCR)-mediated proliferation and cytokine production by CD4+ T cells [18], we also measured the effect of GluIIβ knockout on PD-L2 expression. Figure 2 shows expression of the selected genes in relation to 18s rRNA. Relative expression of the non-target transfected cells was assigned as 100 for all the genes. Of 9 genes chosen according to KEGG enrichment analysis, 8 genes were confirmed to be significantly decreased in all 3 GluIIβ knockout clones, which included PD-L1, VCAN, CDH2, CDH4, ITGA4, ESAM, NECTIN3 and CADM1. Expression of PTPRF was significantly decreased only in 1 out of 3 knockout clones. Further investigation also showed that knockout of GluIIβ also decreased PD-L2 gene expression in cancer cells. Interestingly, the cell morphology of all 3 clones of GluIIβ knockout cells were different from the parental A549 cells and non-targeted transfected cells with less expansion of the cytoplasmic and cell surface area (Fig. 3A and B).

### Proliferation and tumor cell lysing activity of Jurkat E6.1 T cells and PBMCs co-cultured with GluIIß knockout A549 cell

**Fig. 4:**
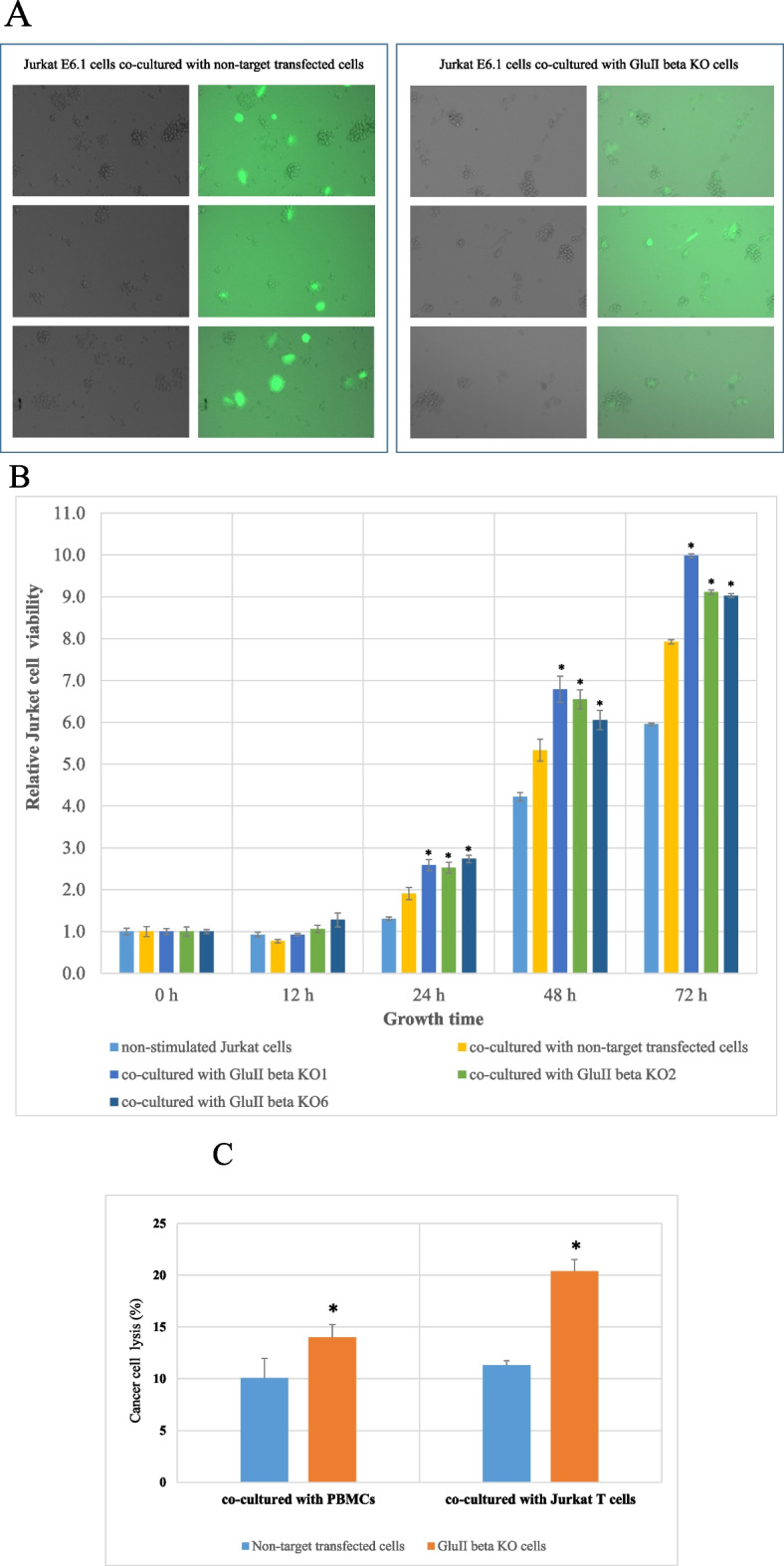
Fluorescence images showing the green fluorescence (GFP) expressing cells, GluIIß KO A549 cells and non-target transfected cells, surrounded by Jurkat E6.1 T cells in the co-culture experiment (**A**). Viability and proliferation of Jurkat E6.1 T cells after being exposed to GluIIß KO A549 cells or non-target control cells for 24 hours as measured at 0, 12, 24, 48 and 72 hours using alamarBlue^®^ (**B**). Cancer cell lysing activity of Jurkat E6.1 T cells and PBMCs co-cultured with GluIIß KO A549 cells compared to those co-cultured with non-target transfected cells (**C**). Bar graphs represent means and standard deviations (SDs) from three independent experiments. *Significant different from those co-cultured with non-target transfected cells (*p<*0.05 by Mann Whitney U test)

The effect of GluIIß knockout cancer cells and non-target transfected cells on viability, proliferation and function of immune cells was investigated utilizing Jurkat E6.1 T cells and PBMCs from healthy volunteers. Jurkat E6.1 T cells or PBMCs were activated with anti-CD3 and anti-CD28 antibodies for 24 hours and subsequently co-cultured with GluIIß knockout, non-target transfected cells. Figure 4A shows green fluorescent of GFP expressing A549 cells surrounded by Jurkat E6.1 T cells in the co-cultured condition. After 24 hours, Jurkat E6.1 T cells were isolated from cancer cells and cell viability was measured at different time points. Figure 4B shows that relative cell number cell viability and proliferation rate of Jurkat E6.1 T cells after stimulated with knockout GluIIß knockout A549 cells exhibited a higher proliferation rate than those co-cultured with non-target transfected cells.

Tumor cell lysing activity of Jurkat E6.1 T cells and PBMCs was also measured after 72 hours of co-culturing. Culture media was collected and subjected to the assessment of LDH. The result showed that Jurkat E6.1 T cells and PBMCs stimulated with GluIIß knockout A549 cells exhibited a better tumor lysing activity than those co-cultured with non-target transfected cells (Fig. 4C).

### Analysis of cytokines released from Jurkat E6-1 T lymphocytes co-cultured with GluIIß knockout A549 cells

**Fig. 5:**
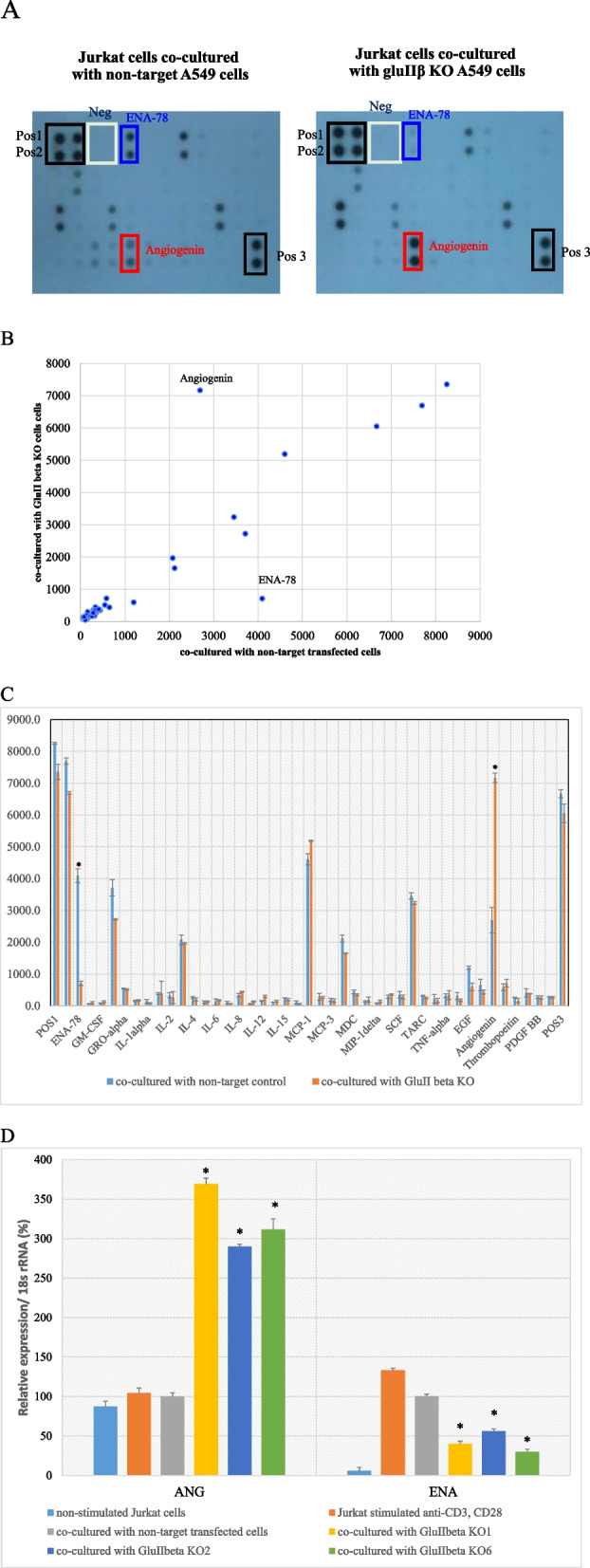
Analysis of secreted cytokines in media of Jurkat E6.1 cells co-cultured with GluIIß knockout cells compared to those co-cultured with non-target transfected cells. Image of cytokine array signals containing 42 different anti-cytokine antibodies spotted in duplicate, including 3 positive and 2 negative controls, hybridized with co-culture media (**A**). Array signals were quantified using “ImageJ software with the Protein Array Analyzer plugin16”. Values of quantified cytokine signals from duplicate spots were averaged and plotted as scatter plot (**B**) with mean values from Jurkat E6.1 cells co-cultured control cells as X-axis and those from Jurkat E6.1 cells co-cultured with KO cells as Y-axis. Mean and standard deviation of quantified signals of each cytokine were plotted as bar graph (**C**) Verification of angiogenin and ENA-7expression level by real time RT-PCR (**D**) *Significant different from those co-cultured with non-target transfected cells (*p<*0.05 by Mann Whitney U test)

After Jurkat E6.1 T cells were treated with anti-human CD3 antibodies and anti-human CD28 antibodies for 24 hours to activate co-stimulatory signal for T cell receptor (TCR). Activated Jurkat E6.1 T cells were then stimulated with GluIIß knockout or non-target transfected A549 cells for 72 hours and cytokines secreted into culture media were determined using Human Cytokine Antibody Array that spotted with antibodies specific for 42 different cytokines in duplicate. Figure 5A shows representative of cytokine array signals from Jurkat E6.1 T cells co-cultured with GluIIß knockout in comparison to those co-cultured with non-target transfected cells. Each membrane contains 3 duplicates of positive control which Fig. 5A shows similar intensity in both membranes and 1 duplicate of negative control which Fig. 5A shows no visible signal.

Signal intensities of each cytokine were quantified using “ImageJ software with the Protein Array Analyzer plugin16”. Quantification values of the duplicate spots of each cytokine were averaged and plotted as scatter plot (Fig. 5B) and bar graph (Fig. 5C). Scatter plot of quantified values from Jurkat E6.1 T cells co-cultured with GluIIß knockout A549 on Y-axis and those co-cultured with non-target transfected cells on X-axis shows that most of the spots were aligned with the 45-degree line, with only 2 spots identified to be angiogenin and ENA-78 deviated from the 45-degree line. Bar graph showed that angiogenin level was significantly increased while ENA-78 level was significantly decreased in culture media from Jurkat E6.1 T cells co-cultured with GluIIß knockout compared to those co-cultured with non-target transfected cells. The verification of the cytokine array results with real time RT-PCR confirmed the same alterations of angiogenin and ENA-78 level in all three clones of GluIIß knockout cells compared to control cells (Fig. 5D).

## Discussion

In this study we have demonstrated for the first time that GluIIß can modulate cellular adhesion molecules (CAMs) of cancer cells. CAMs are a subset of cell surface proteins involved in facilitating the binding of cells to other cells or to the extracellular matrix (ECM), which has been shown to play a critical role in tumor invasion [19]. Knockout of GluIIß caused lung cancer cells to express reduced levels of genes encoded for cellular adhesion molecules and extracellular matrix thus causing an observable change of cell morphology. During tumor development, a reversible transcriptional program named epithelial-mesenchymal transition (EMT) has been shown to be crucial for metastasis, chemoresistance and stemness properties of tumor [20]. One of the hallmarks of EMT is the cadherin switching characterized by the loss of E-cadherin (CDH1) expression and increased expression of N-cadherin (CDH2) [21]. We have shown in this study that knockout of GluIIβ from lung cancer cell line significantly decreased the expression of CDH2 as well as the reduction of many other cell surface proteins including CDH4, PD-L1 (B7-H1) and PD-L2 (B7-DC). PD-L1 and PD-L2 are binding ligands of programmed cell death protein 1 (PD-1) on T cells known to be critical for the negative regulation of T cell’s function [22, 18]. Tumor cells expressing PD-L1, or PD-L2, that can interact with PD-1 will suppress T cell receptor-mediated cytotoxicity and CD8+ T cell proliferation, thus avoiding the killing effect of the autoimmune system and escaping from immune surveillance [23, 24, 18]. GluIIß knockout cancer cells expressed a reduced level of PD-L1 and PD-L2, suppression of GluIIß may thus represent a new approach of revitalizing anti-tumor immunity.

Classic view considers CAMs as tumor suppressors that exert their suppressive effect mainly through cell-adhesion-mediated contact inhibition [25]. Nevertheless, emerging evidence supports the role of CAMs both as a tumor suppressor and a tumor promoter. As adhesion molecules on the cell surface, CAMs is able to modulate the signaling activity of growth factor receptor, which is of great influence on cancer progression. CDH2 has been demonstrated to trigger autophosphorylation of FGFR and the activation of downstream growth signaling independently of ligand [26]. CDH2 was also shown to increase level FGFR by preventing its ubiqitination and degradation [27]. Both FGFR and CDH2 are often overexpressed in metastatic cells [26] and their interaction has been shown to the drive stemlike properties and EMT [28]. Our previous studies have shown that lung cancer tissues exhibited an increased level of GluIIß compared to normal adjacent tissues [11] and hypothesize that this induction caused the cells to become resistant to death pathways as knockout of GluIIß caused cancer cell to undergo autophagy and/or apoptosis [13]. Knockout of GluIIß has also been demonstrated to inhibit growth and metastatic potential of lung cancer cell line [29]. The overall reduction of CAMs in GluIIß knockout cells may thus help explain the underlying mechanism of GluIIß overexpression in promoting tumor growth and progression as well as the suppression of anti-tumor immunity in its microenvironment through the overexpression of genes encoded for CAMs.

Co-culturing of immune cells with GluIIß knockout A549 cells significantly increased proliferation and tumor lysing ability of Jurkat E6.1 T cells and PBMCs compared to those co-culturing with parental or non-target transfected cells. Jurkat E6.1 T cells were utilized as a surrogate for immune cells in this study due to their representation of T lymphocytes, which play a crucial role in the immune response against cancer [30]. The activation and dysfunction of T cells are intricately regulated by receptors, including the PD-1 and PD-L1 pathway, a pathway affected by GluIIß knockout. However, it is essential to acknowledge that Jurkat E6.1 T cells, being an established cell line, may not fully mirror the behavior of actual immune cells. Therefore, to comprehensively assess the impact of GluIIß knockout on immune responses, our investigations were extended to include peripheral blood mononuclear cells (PBMCs). PBMCs encompass a diverse array of immune cell types, mimicking the in vivo environment, thus providing a comprehensive assessment of the anti-tumor immune reactions compared to isolated cell types like macrophages or NK cells. Our results showed that knockout of GluIIß in cancer cells was able to increase the proliferation and tumor lysing ability of both Jurkat E6.1 T cells and PBMCs.

Analysis of secreted cytokines released into the co-culturing media showed significant induction of angiogenin while the level of ENA-78 was significantly decreased in GluIIß knockout cells compared to non-target transfected cells. Angiogenin, also known as ribonuclease 5, is a protein encoded from *ANG* gene that helps increase protein synthesis and cellular proliferation under growth conditions. Nevertheless, during stress conditions, angiogenin translocate to the cytoplasm and cleaves transfer RNA (tRNA) to produce tRNA-derived, stress-induced small RNAs (tiRNAs) that reduce global protein synthesis but induce the translation of anti-apoptotic factors. Although angiogenin has been shown to promote tumor growth and angiogenesis [31], its upregulation in the alloreactive immune response has been shown to inhibit apoptosis of CD4+ T cells [32]. During the adaptive immune response, the expansion phase of T lymphocyte mediated by cell proliferation is followed by the contraction phase through apoptosis. It is possible that an increased level of angiogenin in culture media of Jurkat E6.1 T cells exposed to GluIIß knockout A549 cells during co-culturing helped inhibit apoptosis of T cells and consequently caused and increase of cell viability and tumor lysing activity of T cells compared to those co-culture with control A549 cells.

Analysis of co-cultured media between Jurkat E6.1 T cells and GluIIb KO A549 cells also showed significantly decreased level of ENA-78, also known as CXCL5, compared to those cocultured with A549 control cells. ENA-78, a member of the CXC chemokine family, is identified as an inflammatory mediator with powerful role in neutrophil chemotaxis [33] and generally expressed in monocytes, platelets, endothelial cells, and mast cells. ENA-78 has been reported to be upregulated and associated with tumor progression in many types of cancer (reviewed in [34]). Serum level of ENA-78 was reported to increase in patients with non-small cell lung cancer compared to in healthy controls [35]. An investigation in gastric cancer demonstrated that ENA-78 promoted cancer metastasis through the induction of EMT [36]. A reduction of ENA-78 secretion in GluIIb KO cells thus provides supporting evidence that GluIIβ may involve with the regulation of EMT process and knockout of GluIIb encoding gene help suppression this process.

In conclusion, we have demonstrated in this study that suppression of GluIIß expression in non-small cell lung carcinoma A549 cells affected expression level of genes encoded for cellular adhesion molecules and increased cell viability and tumor-lysing activity of co-cultured Jurkat E6.1 T cells and PBMCs thus suppression of GluIIß in cancer cells may represent a novel approach of boosting anti-tumor activity of immune cells.

### Supplementary Information

**Additional file 1:** **Figure 1A.** Western blot analysis showing GluIIß expression levels in GluIIß KO and non-target transfected cells. **Figure 5A. **Image of cytokine array signals containing 42 different anti-cytokine antibodies spotted in duplicate, including 3 positive and 2 negative controls, hybridized with co-culture media. **Supplementary table 1.** List of primers used in this study.

## Data Availability

The data that support the findings of this study are available from the corresponding author, [RC], upon reasonable request.

## Abbreviations

EGF: Epidermal growth factor
ER: Endoplasmic reticulum
ENA-78: Epithelial-neutrophil activating peptide
G-CSF: Granulocyte-colony-stimulating factor
GluIIβ: Glucosidase II beta subunit
GO: Gene ontology
GRO: Growth-regulated oncogene
IGF-1: Insulin like growth factor 1
IL: Interleukin
IL13RA2: Interleukin-13 receptor subunit alpha-2
IFN: Interferon
KEGG: Kyoto encyclopedia of genes and genomes
KO: Knockout
MCP: Monocyte chemoattractant protein
MCSF: Macrophage colony-stimulating factor
MDC: Macrophage-derived chemokine
MIG: Monokine induced by interferon-gamma
MIP-1δ: Macrophage inflammatory protein-1 delta
PALM2: Paralemmin 2
PDGF-BB: Platelet derived growth factor-BB
PCDHB13: Protocadherin Beta 13
RANTES: Regulated on activation, normal T cell expressed and secreted
SCF: Stem cell factor
SDF-1: Stromal cell-derived factor-1
TARC: Thymus and activation regulated chemokine
TGF: Transforming growth factor
TNF: Tumor necrosis factor
UNC5A: Unc-5 Netrin Receptor A
UPR: Unfolded protein response
VEGF: Vascular endothelial growth factor
ZFPM2: Zinc Finger Protein, FOG Family Member 2
