# Supplementary material for: Transcriptomic analysis of glucosidase II beta subunit (GluIIß) knockout A549 cells reveals its roles in regulation of cell adhesion molecules (CAMs) and anti-tumor immunity
Source: BMC Genomics. 2024 Jan 20;25:82. doi: 10.1186/s12864-023-09888-z (PMC10799456; doi:10.1186/s12864-023-09888-z)
Supplement: Supplementary file 1 — Additional file 1: Figure 1A. Western blot analysis showing GluIIß expression levels in GluIIß KO and non-target transfected cells. Figure 5A. Image of cytokine array signals containing 42 different anti-cytokine antibodies spotted in duplicate, including 3 positive and 2 negative controls, hybridized with co-culture media. Supplementary table 1. List of primers used in this study. [file 12864_2023_9888_MOESM1_ESM.pdf]

Figure 1A: Western blot analysis showing GluIIß expression levels in GluIIß KO and non-target transfected cells

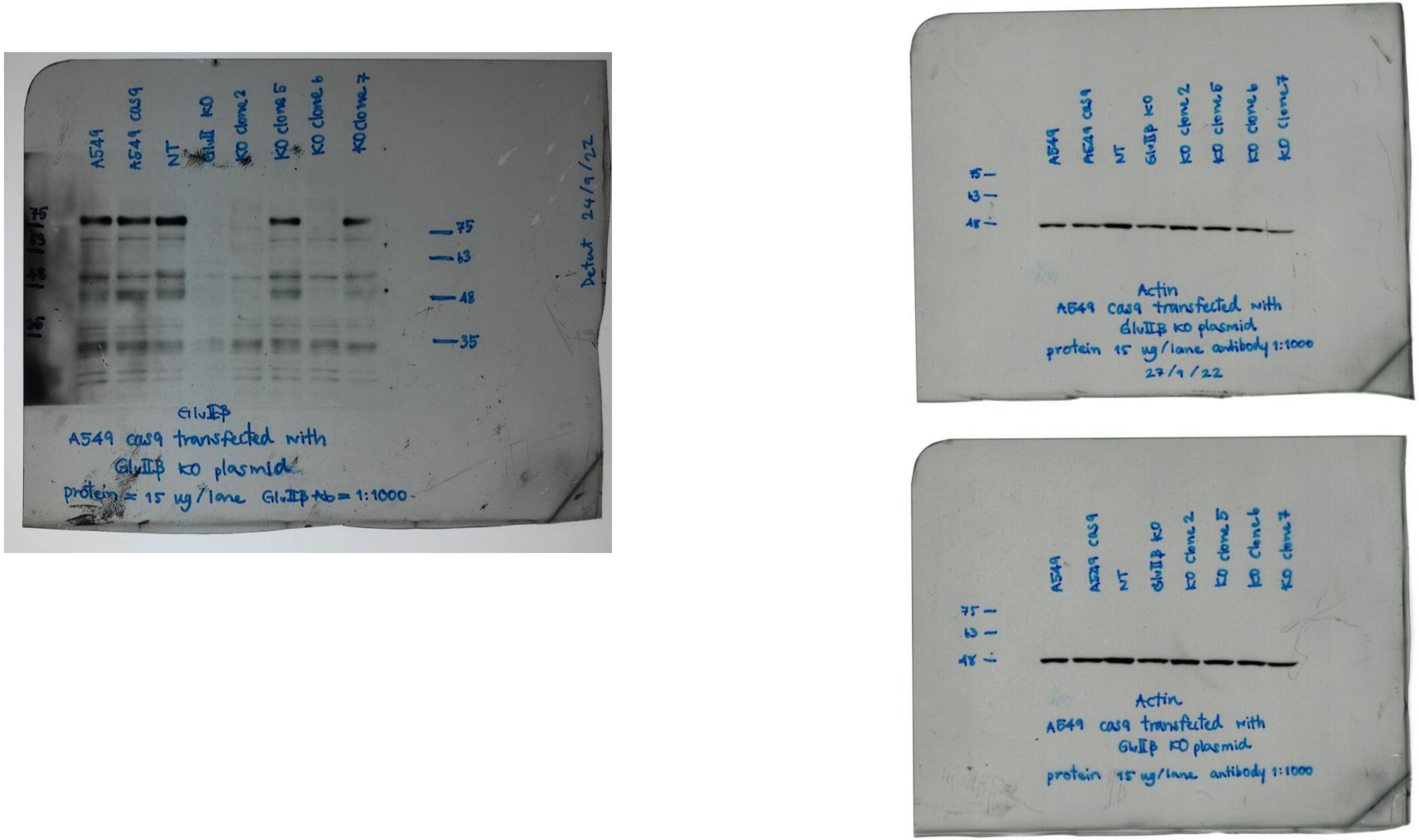

Western blot signals form anti-actin antibodies showed single-sized bands around 45Kd (expected size of actin), therefore, no longer time film exposure was carried out

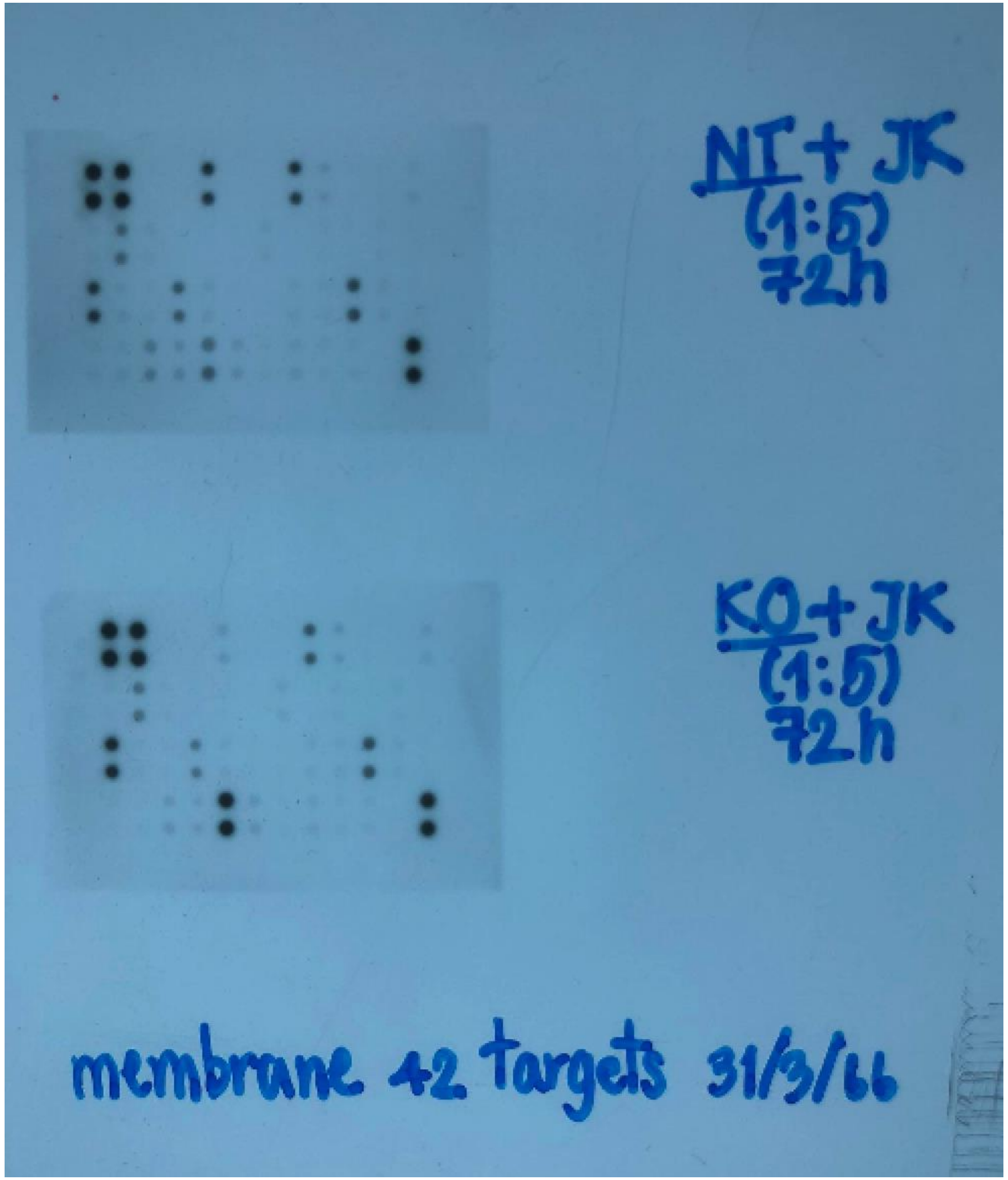

Figure 5A Image of cytokine array signals containing 42 different anti-cytokine antibodies spotted in duplicate, including 3 positive and 2 negative controls, hybridized with co-culture media.

Supplementary table 1. List of primers used in this study

Gene name	Forward	Reverse
PD-L1 [1]	5'- AAATGGAACCTGGCGAAAGC -3'	5'- GATGAGCCCCTCAGGCATTT -3'
VCAN [2]	5′- AGGATACAGCGGAGACCAGT -3'	5′- GAAGGCAGAGGCACCTGAAT - 3′
CDH2 [3]	5′- GCCCCTCAAGTGTTACCTCAA -3′	5′- AGCCGAGTGATGGTCCAATTT -3′
CDH4 [4]	5′- CACCAAAAACAACGTCTACGAG -3′	5′- GAAGACCAGCAGGGAGTCATAG -3′
PTPRF [5]	5′- ATGTCATCGCCTACGACCACTC -3′	5′- GTGGCGATGTAGGCATTCTGCT -3′
IGTA4 [6]	5′- TCGGAGCCAGCATACTACC -3	5′- CCACAGCACAGACAGAAGC -3′
ESAM [7]	5'- ATAGAATTCATGATTTCCCTCCCGGGGC -3'	5'- TTAGAATTCTCATACCAGAGAGCCGCTTGAC -3'
NECTIN3 [8]	5'- CACCTCCGCTGCTGCTGCTG CTCTTCCC -3'	5'- CAGTTGTAGAGGACTGGGCATTTCC -3'
CADM1 [9]	5'- CCACAGGTGATGGGCAGAAT -3'	5'- TTCCTGTGGGGGATCGGTAT -3'
PD-L2 [10]	5'- GTCTTGGGAGCCAGGGTGAC -3'	5'- TGAAAAGTGCAAATGGCACGC -3'
ENA-78 [11]	5'-CGGGAAGGAAATTTGTCTTGA-3'	5'-AGCTTAAGCGGCAAACATAGG-3'
Angiogenin [12]	5'-AGAAGCGGGTGAGAAACAA-3'	5'-CTTCCAACACAGGCTCCTCG-3'
18s-rRNA	5′-AGGAATTGACGGAAGGGCAC-3′	5′-GTGCAGCCCCGGACATCTAAG-3′

References for primers

1. Wu H, Zhu B, Shimoishi Y, Murata Y, Nakamura Y: (-)-Epigallocatechin-3-gallate induces up-regulation of Th1 and Th2 cytokine genes in Jurkat T cells. *Arch Biochem Biophys* 2009, 483(1):99-105.
2. Cheng Y, Sun H, Wu L, Wu F, Tang W, Wang X, Lv C: VUp-Regulation of VCAN Promotes the Proliferation, Invasion and Migration and Serves as a Biomarker in Gastric Cancer. *Onco Targets Ther* 2020, 13:8665-8675.
3. Yang H, Wang L, Zhao J, Chen Y, Lei Z, Liu X, Xia W, Guo L, Zhang HT: TGF-beta-activated SMAD3/4 complex transcriptionally upregulates N-cadherin expression in non-small cell lung cancer. *Lung Cancer* 2015, 87(3):249-257.
4. Li Z, Su D, Ying L, Yu G, Mao W: Study on expression of CDH4 in lung cancer. *World J Surg Oncol* 2017, 15(1):26.
5. Tian X, Yang C, Yang L, Sun Q, Liu N: PTPRF as a novel tumor suppressor through deactivation of ERK1/2 signaling in gastric adenocarcinoma. *Onco Targets Ther* 2018, 11:7795-7803.
6. Mostafavi-Pour Z, Ashrafi MR, Talaei-Khozani T: Down regulation of ITGA4 and ITGA5 genes after formation of 3D spherules by human Wharton's jelly stem cells (hWJSCs). *Mol Biol Rep* 2018, 45(3):245-252.
7. Kimura R, Ishida T, Kuriyama M, Hirata K, Hayashi Y: Interaction of endothelial cell-selective adhesion molecule and MAGI-1 promotes mature cell-cell adhesion via activation of RhoA. *Genes Cells* 2010, 15(4):385-396.
8. Cocchi F, Menotti L, Di Ninni V, Lopez M, Campadelli-Fiume G: The herpes simplex virus JMP mutant enters receptor-negative J cells through a novel pathway independent of the known receptors nectin1, HveA, and nectin2. *J Virol* 2004, 78(9):4720-4729.
9. Huang Y, Feng G: MiR-423-5p aggravates lung adenocarcinoma via targeting CADM1. *Thorac Cancer* 2021, 12(2):210-217.
10. Hassan SS, Akram M, King EC, Dockrell HM, Cliff JM: PD-1, PD-L1 and PD-L2 Gene Expression on T-Cells and Natural Killer Cells Declines in Conjunction with a Reduction in PD-1 Protein during the Intensive Phase of Tuberculosis Treatment. *PLoS One* 2015, 10(9):e0137646.
11. Zineh I, Beitelshees AL, Welder GJ, Hou W, Chegini N, Wu J, Cresci S, Province MA, Spertus JA: Epithelial neutrophil-activating peptide (ENA-78), acute coronary syndrome prognosis, and modulatory effect of statins. *PLoS One* 2008, 3(9):e3117.
12. Yang H, Yuan L, Ibaragi S, Li S, Shapiro R, Vanli N, Goncalves KA, Yu W, Kishikawa H, Jiang Y *et al*: Angiogenin and plexin-B2 axis promotes glioblastoma progression by enhancing invasion, vascular association, proliferation and survival. *Br J Cancer* 2022, 127(3):422-435.
